# A combined lipidomic and 16S rRNA gene amplicon sequencing approach reveals archaeal sources of intact polar lipids in the stratified Black Sea water column

**DOI:** 10.1111/gbi.12316

**Published:** 2018-10-03

**Authors:** Martina Sollai, Laura Villanueva, Ellen C. Hopmans, Gert‐Jan Reichart, Jaap S. Sinninghe Damsté

**Affiliations:** ^1^ Departments of Marine Microbiology and Biogeochemistry and Ocean Systems NIOZ Royal Netherlands Institute for Sea Research and Utrecht University Den Burg The Netherlands; ^2^ Department of Earth Sciences Faculty of Geosciences University of Utrecht Utrecht The Netherlands

**Keywords:** archaeal communities, archaeol, biogeochemical processes, biomarkers, element cycles, glycerol dialkyl glycerol tetraethers, intact polar lipids, microbial ecology

## Abstract

Archaea are important players in marine biogeochemical cycles, and their membrane lipids are useful biomarkers in environmental and geobiological studies. However, many archaeal groups remain uncultured and their lipid composition unknown. Here, we aim to expand the knowledge on archaeal lipid biomarkers and determine the potential sources of those lipids in the water column of the euxinic Black Sea. The archaeal community was evaluated by 16S rRNA gene amplicon sequencing and by quantitative PCR. The archaeal intact polar lipids (IPLs) were investigated by ultra‐high‐pressure liquid chromatography coupled to high‐resolution mass spectrometry. Our study revealed both a complex archaeal community and large changes with water depth in the IPL assemblages. In the oxic/upper suboxic waters (<105 m), the archaeal community was dominated by marine group (MG) I Thaumarchaeota, coinciding with a higher relative abundance of hexose phosphohexose crenarchaeol, a known marker for Thaumarchaeota. In the suboxic waters (80–110 m), MGI *Nitrosopumilus* sp. dominated and produced predominantly monohexose glycerol dibiphytanyl glycerol tetraethers (GDGTs) and hydroxy‐GDGTs. Two clades of MGII Euryarchaeota were present in the oxic and upper suboxic zones in much lower abundances, preventing the detection of their specific IPLs. In the deep sulfidic waters (>110 m), archaea belonging to the DPANN Woesearchaeota, Bathyarchaeota, and ANME‐1b clades dominated. Correlation analyses suggest that the IPLs GDGT‐0, GDGT‐1, and GDGT‐2 with two phosphatidylglycerol (PG) head groups and archaeol with a PG, phosphatidylethanolamine, and phosphatidylserine head groups were produced by ANME‐1b archaea. Bathyarchaeota represented 55% of the archaea in the deeper part of the euxinic zone and likely produces archaeol with phospho‐dihexose and hexose‐glucuronic acid head groups.

## 1 INTRODUCTION

Archaea form a broad and diverse domain with a complex phylogeny. The recognition of new phyla by the application of culture‐independent approaches is substantially contributing to this still increasing complexity (e.g., Castelle et al., 2015; Iverson et al., 2012; Spang et al., 2015). The marine water column and sediments have been shown to be a niche for complex archaeal communities. Some of the archaea comprising these communities have been successfully cultivated providing clues on their physiology. For example, marine Thaumarchaeota (previously known as marine group I [MGI]; Brochier‐Armanet, Boussau, Gribaldo, & Forterre, 2008; Spang et al., 2010) are aerobic ammonia oxidizers commonly found in epipelagic and mesopelagic water column and in sediments (Francis, Roberts, Beman, Santoro, & Oakley, 2005; Könneke et al., 2005; Venter et al., 2004; Wuchter et al., 2006). Other archaeal planktonic lineages include euryarchaeotal marine group II (MGII) (DeLong, 1992; Fuhrman, McCallum, & Davis, 1992) and the closely related marine group III (MGIII) (Fuhrman et al., 1992). MGII are predicted to be motile photoheterotrophs based on genome analysis (Iverson et al., 2012) but remain uncultured. Members of the MGII reside mainly in the marine photic zone although other ecotypes have been detected in deeper water columns (see Zhang, Xie, Martin‐Cuadrado, & Rodriguez‐Valera, 2015 for a review). MGIII archaea have been mainly detected in deep mesopelagic and bathypelagic, and most recently also in epipelagic waters, but their physiology is essentially unknown (Bano, Ruffin, Ransom, & Hollibaugh, 2004; Haro‐Moreno, Rodriguez‐Valera, López‐García, Moreira, & Martin‐Cuadrado, 2017; López‐García, Moreira, López‐López, & Rodriguez‐Valera, 2001; Massana, DeLong, & Pedros‐Alio, 2000), also because they remain uncultured. In addition, the presence of members of the archaeal superphylum DPANN in marine settings, mainly in microbial mats, sediments, plankton, and hydrothermal vents, is increasingly reported (Pachiadaki, Kallionaki, Dählmann, De Lange, & Kormas, 2011; Robertson, Spear, Harris, & Pace, 2009). Studies based on genome analysis have concluded that members of this superphylum lack important metabolic pathways, suggesting that they have a symbiotic or parasitic lifestyle (Castelle et al., 2015; Rinke et al., 2013).

In marine sediments, other yet‐uncultured archaeal groups have been detected. For example, members of the anaerobic methanotrophic archaea (ANME), performing the anaerobic oxidation of methane (AOM), were firstly identified in shallow marine anoxic sediments rich in methane hydrates (Boetius et al., 2000; Hinrichs, Hayes, Sylva, Brewer, & DeLong, 1999; Orphan et al., 2001; Pancost et al., 2000). ANME have also been detected in the anoxic water column based on specific membrane lipids (Schouten, Wakeham, & Sinninghe Damsté, 2001; Wakeham, Lewis, Hopmans, Schouten, & Sinninghe Damsté, 2003) and, subsequently, based on genomic data (Vetriani, Tran, & Kerkhof, 2003). Archaeal benthic groups include the Marine Benthic Group B (MBG‐B), the Marine Benthic Group D (MBG‐D), and the phylum Bathyarchaeota (formerly known as the Miscellaneous Crenarchaeotal Group, MCG) (Inagaki et al., 2003; Spang et al., 2015; Vetriani & Jannasch, 1999), which often co‐occur, supposedly living as heterotrophs consuming buried recalcitrant organic matter, degrading proteins, and lipids (Biddle et al., 2006; Kubo et al., 2012; Lloyd et al., 2013; Meng et al., 2014). More recently, lineages of the Bathyarchaeota phylum have been suggested to be homoacetogens, as well as involved in methane metabolism, based on genomic and enzymatic evidence (Evans et al., 2015; He et al., 2016).

Despite the huge effort of molecular ecologists to untangle archaeal diversity in the marine environment, many aspects of archaeal physiology, abundance, and preferred niche still need to be addressed. The employment of combined approaches may assist in overcoming the potential limitations due to the lack of cultivated representatives and the intrinsic biases of many DNA‐based techniques (Marine et al., 2014; Pinto & Raskin, 2012; Teske & Sørensen, 2008). A complementary method of investigation is supplied by lipidomics. Targeting microbial lipids (Schouten et al., 2008; Sturt, Summons, Smith, Elvert, & Hinrichs, 2004) may offer insight in addition to PCR‐based approaches avoiding potential biases although the lipids commonly lack the taxonomic resolution that DNA‐based techniques can offer. Some lipids, however, have been shown to be specific of certain microbial groups and are used as taxonomic biomarkers of their presence in the environment (e.g., Hamersley et al., 2007; Rush, Wakeham, Hopmans, Schouten, & Sinninghe Damsté, 2012; Sinninghe Damsté, Schouten, Hopmans, van Duin, & Geenevasen, 2002). Lipid molecules can be preserved much longer than nucleic acids in the sedimentary record, providing information on past microbial communities (Castañeda & Schouten, 2011; Eglinton & Eglinton, 2008; Kuypers et al., 2001).

Archaeal membranes are formed by unique core lipids (CL), specifically phytanyl glycerol diether (archaeol) and glycerol dibiphytanyl glycerol tetraethers (GDGTs). GDGTs may contain up to eight cyclopentane moieties (see Supporting Information Figure S1 for structures); many studies have shown they are generally nonspecific and occur in a wide range of archaea such as extremophiles (Bauersachs, Weidenbach, Schmitz, & Schwark, 2015; Ellen et al., 2009; Schouten et al., 2007), methanogens (Bauersachs et al., 2015; Koga, Akagawa‐Matsushita, Ohga, & Nishihara, 1993), ANMEs (Blumenberg, Seifert, Reitner, Pape, & Michaelis, 2004; Pancost, Hopmans, & Sinninghe Damsté, 2001; Wakeham, Hopmans, Schouten, & Sinninghe Damsté, 2004; Wegener, Krukenberg, Ruff, Kellermann, & Knittel, 2016), and Thaumarchaeota (Elling et al., 2017; Pitcher, Villanueva et al., 2011; Schouten et al., 2008; Sinninghe Damsté et al., 2002). To date, crenarchaeol, the GDGT containing four cyclopentane moieties and a cyclohexane moiety (Sinninghe Damsté et al., 2002), represents a unique case being considered characteristic of Thaumarchaeota (Schouten et al., 2008; Sinninghe Damsté et al., 2012). Knowledge on the membrane lipid composition of archaeal groups is based on the availability of cultures, but some recent studies have inferred the lipid membrane composition based on correlations between a specific lipid and 16S rRNA gene reads of a specific archaeal group. For example, butanetriol dibiphytanyl glycerol tetraether lipids (BDGTs; Supporting Information Figure S1) have been attributed to archaea of the Miscellaneous Crenarchaeotic Group (MCG) (Meador et al., 2015), although they were recently also found in a methanogen (Becker et al., 2016). This approach also led to the suggestion that MGII archaea synthesize GDGTs including crenarchaeol (Lincoln et al., 2014a), and unsaturated archaeol with 0–4 double bonds (Zhu et al., 2016). However, the limited characterization of the taxonomic composition of the analyzed samples and differences in the resilience times between CL‐GDGTs (attributed to dead biomass) and DNA can jeopardize the association of lipid biomarkers with their potential biological sources (e.g., Lincoln et al., 2014b; Schouten, Villanueva, Hopmans, van der Meer, & Sinninghe Damsté, 2014).

An important breakthrough for lipid‐based studies has been the development of methods that allow to detect archaeal intact polar lipids (IPLs), which are composed of the core lipid attached to one or two polar headgroups (Sturt et al., 2004). These headgroups are rapidly released by hydrolysis once the cell dies, and consequently, IPLs are considered as biomarkers of living biomass. The phosphate‐ester bond has been experimentally proven to be especially labile (Harvey, Fallon, & Patton, 1986; White, Davis, Nickels, King, & Bobbie, 1979). Consequently, the IPL hexose phosphohexose (HPH) crenarchaeol is considered as an excellent biomarker of living Thaumarchaeota (Lengger et al., 2012; Schouten, Middelburg, Hopmans, & Sinninghe Damsté, 2010; Schouten et al., 2012). However, such instances are rare and due to the difficulties in cultivating the predominant archaea in the marine environment, the need for new reliable archaeal biomarkers is critical to study these microorganisms in the environment and to be able to use this information to investigate the sedimentary record.

This study is aimed at expanding the array of known archaeal IPLs in the marine environment and to provide clues on the archaeal groups as potential sources of IPLs. To this end, we analyzed the archaeal diversity based on 16S rRNA gene amplicon sequencing, as well as the archaeal IPL composition by ultra‐high‐pressure liquid chromatography coupled to high‐resolution mass spectrometry (UHPLC‐HRMS) in the Black Sea water column using a comprehensive approach. Previous Black Sea studies (e.g., Coolen et al., 2007; Kuypers et al., 2003; Schubert et al., 2006) have used combined DNA/lipid approaches but were targeted on a specific group of archaea or bacteria. The Black Sea is the largest permanently stratified anoxic basin in the world, and its water column is characterized by the presence of strong redox gradients and therefore represents an excellent setting to target diverse archaeal groups with a comprehensive approach targeting both archaeal DNA and IPLs.

## 2 MATERIALS AND METHODS

### 2.1 Sampling and physicochemical measurements

Sampling was performed during the Phoxy cruise (June–July 2013) aboard of the *R/V Pelagia*. The sampling station (PHOX2) was located at 42°53.8′N and 30°40.7′E in the western gyre of the Black Sea. SPM (water volume 148–796 L) was collected on pre‐ashed 142‐mm‐diameter 0.7‐μm pore size glass fiber GF/F filters (Pall Corporation, Washington) mounted on McLane WTS‐LV in situ pumps (McLane Laboratories Inc., Falmouth). In each cast, three pumps were deployed simultaneously at different depths. During a total of five pumping sessions, SPM from 15 different water depths was obtained. Upon the recovery of the in situ pumps on the deck of the ship, the filters were immediately stored at −80°C. Both IPL‐based characterization and DNA‐based characterization were performed on the same filters, allowing a direct comparison of results.

Physical parameters of the water column were recorded by a conductivity–temperature–density (CTD) unit (SBE 911 plus, Sea‐Bird Electronics). Dissolved oxygen (O_2_) concentrations were measured by a SBE 43 electrochemical sensor mounted on the CTD rosette. The sensor has a detection limit of 1–2 μM, which has been recently proven to overestimate the oxygen level at the lowest concentrations (Tiano et al., 2014). Samples for inorganic nitrogen nutrients (i.e., ${\text{NO}}_{3}^{-}$, ${\text{NO}}_{2}^{-}$, and ${\text{NH}}_{4}^{+}$) and for hydrogen sulfide (HS^‐^) were obtained with a GoFlow rosette sampler (General Oceanics, Miami) from the same water depths sampled for SPM. The water collected in the CTD bottles was immediately processed on‐board, and the concentrations were determined within 18 hr on a QuAAtro autoanalyzer. Specifically, ca. 5 ml samples were filtered over Acrodisc PF (pre‐filter) Syringe Filter with 0.8/0.2 μm Supor membrane (Pall Corporation) into separate pre‐rinsed pony vials. One vial already containing 40 μl 1N NaOH was used for HS^−^ analysis and one without any addition of NaOH for DIC. Another glass vial was used for ${\text{NO}}_{3}^{-}$, ${\text{NO}}_{2}^{-}$, and ${\text{NH}}_{4}^{+}$ analysis. The detection limits for ${\text{NO}}_{3}^{-}$, ${\text{NO}}_{2}^{-}$, and ${\text{NH}}_{4}^{+}$ were 0.008, 0.006, and 0.044 μM, respectively. The detection limit for HS^−^ was 0.263 μmol/L.

### 2.2 DNA‐based analyses

DNA/RNA was extracted from 1/8 (SPM samples from 50 to 110 m depth) or 1/4 (SPM samples from 130 to 2,000 m depth) sections of the GF/F filter with the RNA PowerSoil^®^ Total Isolation Kit plus the DNA elution accessory (Mo Bio Laboratories, Carlsbad, CA). Concentration of DNA was quantified by using Nanodrop (Thermo Scientific, Waltham, MA) and fluorometrically by using Quant‐iT^™^ PicoGreen^®^ dsDNA Assay Kit (Life Technologies, Netherlands).

The 16S rRNA gene amplicon sequencing and analysis were performed with the primers S‐D‐Arch‐0159‐a‐S‐15 and S‐D‐Bact‐785‐a‐A‐21 (Klindworth et al., 2013) using 454 GLX sequencing with a single‐ended approach as described in Moore et al. (2015). The archaeal 16S rRNA gene amplicon sequences were analyzed by qiime v1.9 (Caporaso et al., 2010). Raw sequences were demultiplexed and then quality‐filtered with a minimum quality score of 25, length between 250 and 350, and allowing up to two mismatches in the barcode sequence. OTU picking step was performed with Usearch with a threshold of 0.97 (roughly corresponding to species‐level OTUs). Taxonomy was assigned based on blast and the SILVA database version 123 (Altschul, Gish, Miller, Myers, & Lipman, 1990; Quast et al., 2013). The 16S rRNA gene amplicon reads (raw data) have been deposited in the NCBI Sequence Read Archive (SRA) under BioProject number PRJNA423140.

Amplification of the archaeal *amoA* gene was performed as described by Yakimov et al. (2011) with DNA extracts of SPM obtained at 50, 85, 100, 500, and 2,000 m water depth. The PCR mixture was the following (final concentration): Q‐solution 1× (PCR additive, Qiagen); PCR buffer 1×; BSA (200 μg/ml); dNTPs (20 μM); primers (0.2 pmol/μl); MgCl_2_ (1.5 mM); 1.25 U Taq polymerase (Qiagen, Valencia, CA, USA). PCR conditions were the following: 95°C, 5 min; 35× [95°C, 1 min; 55°C, 1 min; 72°C, 1 min]; final extension 72°C, 5 min. PCR products were gel purified (QIAquick gel purification kit, Qiagen) and cloned in the TOPO‐TA cloning^®^ kit from Invitrogen (Carlsbad, CA, USA) and transformed in *Escherichia coli* TOP10 cells following the manufacturer's recommendations. Recombinant clones plasmid DNAs were purified by Qiagen Miniprep kit and screening by sequencing (total *n *=* *115) using M13R primer by Macrogen Europe Inc. (Amsterdam, The Netherlands). Archaeal *amoA* gene sequences are deposited in the NCBI database with accession numbers MG760455–MG760569. The obtained archaeal *amoA* gene sequences were translated into protein sequences that were aligned with already annotated *amoA* sequences by using the Muscle application (Edgar, 2004). Phylogenetic trees were constructed with the Neighbor‐joining method (Saitou & Nei, 1987) and evolutionary distances computed using the Poisson correction method with a bootstrap test of 1,000 replicates. The *amoA*‐based phylogenetic trees were used to relate the thaumarchaeotal sequences detected in this study with others of the same phylum detected in other settings.

Quantification of archaeal 16S rRNA gene copies was performed by quantitative PCR (qPCR) using the primers Parch519F and Arc915R as previously described (Pitcher, Villanueva et al., 2011). The standard curve was generated by PCR amplifying an insert of an archaeal 16S rRNA gene (as in Pitcher, Hopmans et al., 2011). The qPCR mixture (25 μl) contained 1 Unit of Pico Maxx high‐fidelity DNA polymerase (Stratagene, Agilent Technologies, Santa Clara, CA) 2.5 μl of 10× Pico Maxx PCR buffer, 2.5 μl 2.5 mM of each dNTP, 0.5 μl BSA (20 mg/ml), 0.02 pmol/μl of primers, 10,000 times diluted SYBR Green^®^ (Invitrogen) (optimized concentration), 0.5 μl 50 mM of MgCl_2_, and ultrapure sterile water. All reactions were performed in iCycler iQ™ 96‐well plates (Bio‐Rad, Hercules CA). Specificity of the reaction was tested with a gradient melting temperature assay. The cycling conditions for the qPCR were the following: 95°C, 4 min; 40–45× [95°C, 30 s; 62°C, 40 s; 72°C, 30 s]; final extension 80°C, 25 s. The qPCR products were performed in triplicate with standard curves from 10^0^ to 10^7^ molecules per microliter. qPCR efficiency was 98.6% and *R^2^* = 0.995.

### 2.3 Lipid extraction and analysis

Intact polar lipids (IPLs) were extracted from the GF/F filter using a modified Bligh‐Dyer method and analyzed as described in Sturt et al. (2004) with some modifications. 1‐O‐hexadecyl‐2‐acetyl‐*sn*‐glycero‐3‐phosphocholine (PAF) was added as internal standard to the extracts and dried under a stream of nitrogen. The extracts with the added standard were then dissolved in the injection solvent (hexane:isopropanol:H_2_O 718:271:10 [v/v/v]) and filtered through a 0.45‐μm, 4‐mm‐diameter True Regenerated Cellulose syringe filter (Grace Davison, Columbia, MD, USA). For IPL analysis, an Ultimate 3000 RS UHPLC, equipped with thermostated auto‐injector and column oven, coupled to a Q Exactive Orbitrap MS with Ion Max source with heated electrospray ionization (HESI) probe (Thermo Fisher Scientific, Waltham, MA) was used (see Besseling, Hopmans, Sinninghe Damsté, & Villanueva, 2018 for details). Separation was achieved at 30°C, on an YMC‐Triart Diol‐HILIC column (250 × 2.0 mm, 1.9 μm particles, pore size 12 nm; YMC Co., Ltd, Kyoto, Japan). The elution program employed comprised a flow rate of 0.2 ml/min: 100% A for 5 min, followed by a linear gradient to 66% A: 34% B in 20 min, maintained for 15 min, followed by a linear gradient to 40% A: 60% B in 15 min, followed by a linear gradient to 30% A: 70% B in 10 min, where A = hexane/2‐propanol/formic acid/14.8 M NH_3aq_ (79:20:0.12:0.04 [volume in volume in volume in volume, v/v/v/v]) and B = 2‐propanol/water/formic acid/ 14.8 M NH_3aq_ (88:10:0.12:0.04 [v/v/v/v]). Total run time was 70 min with a re‐equilibration period of 20 min in between runs. HESI settings comprised: sheath gas (N_2_) pressure 35 (arbitrary units), auxiliary gas (N_2_) pressure 10 (arbitrary units), auxiliary gas (N_2_) T 50°C, sweep gas (N_2_) pressure 10 (arbitrary units), spray voltage 4.0 kV (positive ion ESI), capillary temperature 275°C, S‐Lens 70 V. IPLs were analyzed with a mass range of *m*/*z* 375–2,000 with a resolving power of 70,000 followed by data‐dependent MS^2^ (resolution 17,500), in which the ten most abundant masses in the mass spectrum (with the exclusion of isotope peaks) were fragmented successively (stepped normalized collision energy 15, 22.5, 30; isolation window 1.0 *m*/*z*). A dynamic exclusion window of 6 s, with a mass tolerance of 3 ppm, was applied. In order to target‐specific 193 specific IPLs, we used an inclusion list with a mass tolerance of 3 ppm (see Supporting Information Table S2). The Q Exactive was calibrated within a mass accuracy range of 1 ppm using the Thermo Scientific Pierce LTQ Velos ESI Positive Ion Calibration Solution (containing a mixture of caffeine, MRFA, Ultramark 1621, and *N*‐butylamine in an acetonitrile/methanol/acetic acid solution).

Peak areas for each individual IPL were determined by integrating the combined mass chromatogram (within 3 ppm) of the monoisotopic and first isotope peak of all relevant adducts formed (protonated, ammoniated, and/or sodiated adducts may be formed in different proportions depending on the type of IPL). The response of the internal standard PAF was monitored to correct for possible matrix effects and variations in mass spectrometer performance. Reported peak areas have been corrected for these effects. As no authentic standards were available for absolute quantitation of the individual IPLs, the abundances are reported as response units per liter of water (r.u./L).

### 2.4 Statistical analyses

The existence of a linear correlation between the abundances of specific archaeal groups and the IPLs detected in the Black Sea was tested by applying a Pearson correlation analysis, performed with the r software package for statistical computing (http://cran.r-project.org/). Possible spurious correlations were also tested by applying the same statistical analysis among the archaeal lineages detected. The data employed to build the correlation matrices included the total archaeal 16S rRNA gene reads of the different archaeal classes (as determined by amplicon sequencing) detected in the Black Sea SPM at different depths (copies/L) and the abundance of the IPL classes as obtained with the UHPLC‐HRMS analysis of the Black Sea SPM at different depths (expressed as response units per Liter, r.u./L). Quantification of IPLs, however, remains semi‐quantitative due to the lack of authentic standards for structurally different IPLs that may cause variations in the MS signal response factors (Sturt et al., 2004). The correlation was expressed as coefficients (*r* values) ranging from −1 to +1, where negative *r* values indicate a negative linear correlation between the two variables, positive values indicate a positive linear correlation and 0 indicates no correlation.

## 3 RESULTS

We collected suspended particulate matter (SPM) in higher resolution than previous studies (15 depths from 50 to 2,000 m depth) across the water column of the Black Sea at station PHOX2, which is located in the western basin of the Black Sea at 2,179 m water depth in Bulgarian waters (Supporting Information Figure S2). Temperature and salinity measurements were obtained by CTD measurements and allowed calculation of the potential density anomaly (*σ*_θ_) of the water masses. Physicochemical parameters were measured in water collected from the Niskin bottles connected to the CTD rosette (Supporting Information Table S1; Reichart et al., 2013).

### 3.1 Physicochemical conditions in the water column

**Figure 1:**
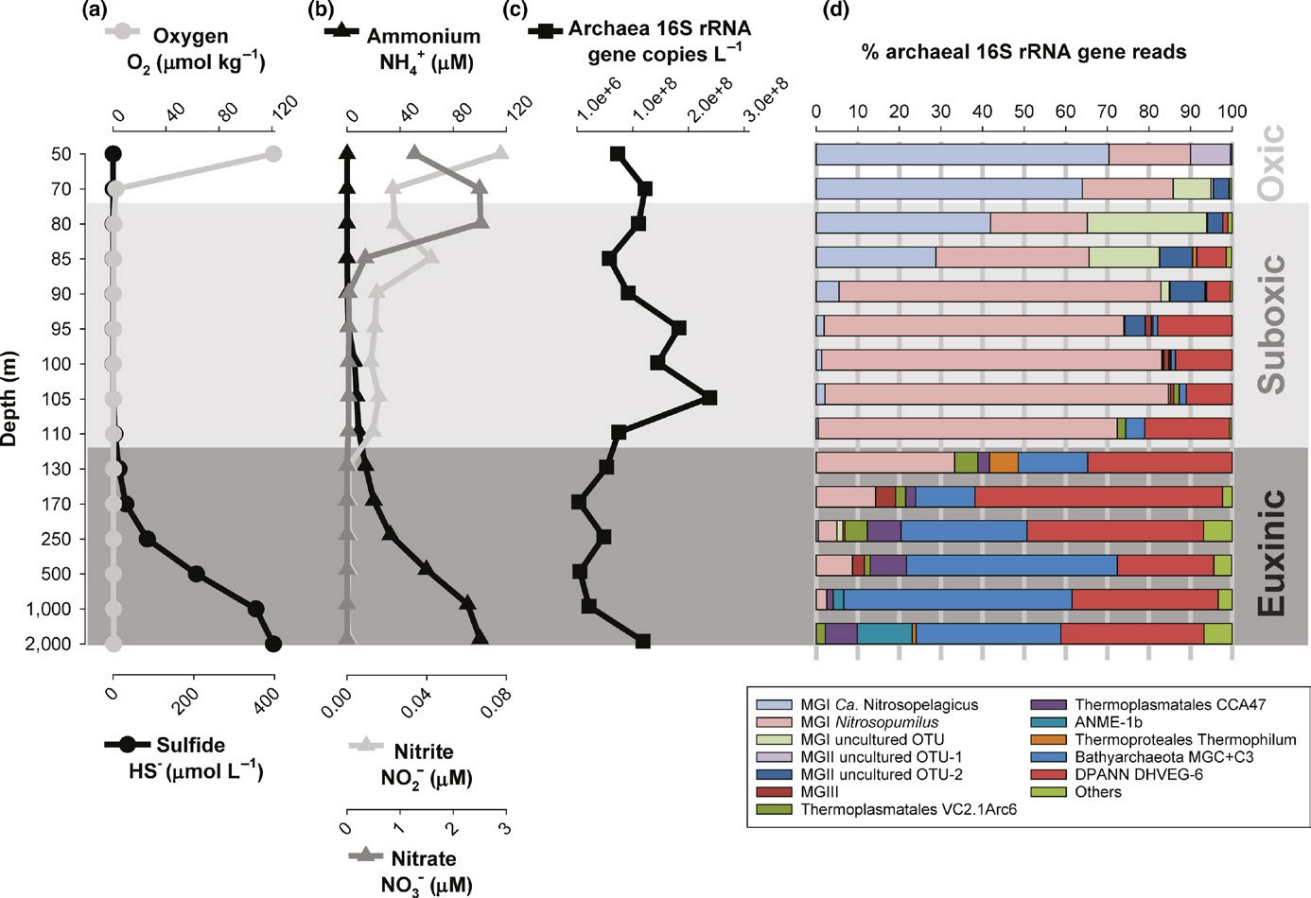
Concentration profiles of (a) oxygen (O_2_, μmol/kg) and sulfide (HS ^−^, μmol/L), (b) ammonia (${\text{NH}}_{4}^{+}$, μM), nitrite (${\text{NO}}_{2}^{-}$, μM), and nitrate (${\text{NO}}_{3}^{-}$, μM). (c) The absolute number of total archaeal 16S rRNA gene (copies/L), and (d) percentage of the archaeal 16S rRNA gene reads of the archaeal groups detected across the water column of the Black Sea at station PHOX2. [Colour figure can be viewed at wileyonlinelibrary.com]

The oxygen concentration was 121 μmol/kg at 50 m depth (*σ*_θ_ ~14.9), decreased to 2.2 μmol/kg at 70 m depth (*σ*_θ_ ~15.7), and to 0.5 μmol/kg at 80 m (*σ*_θ_ ~15.9) (Figure 1a). From 80 to 2,000 m depth, O_2_ was below the limit of detection (0.3 μmol/kg). The sulfide concentration was below the limit of detection up to 100 m depth and was 0.9 μmol/L at 105 m and 4.6 μmol/L at 110 m depth (Figure 1a). From this depth on, the sulfide concentration increased substantially reaching approximately 400 μmol/L at 2,000 m (*σ*_θ_ ~17.2). On the basis of these profiles, we define for this study the “oxic zone” as the 0–75 m depth range, the “suboxic zone” as the 75–115 m range, and the “euxinic zone” as the 115–2,000 m range. Note that the basal part of the thus defined “suboxic zone” contains traces of sulfide (<1.2% of the maximum concentrations). The nitrate concentration was ~1.3 μmol/L at 50 m depth (*σ*_θ_ ~14.9) and reached its maximum (i.e., ~2.5 μmol/L) between 70 and 80 m depth (*σ*_θ_ ~15.7–15.9) (Figure 1b). Subsequently, it gradually decreased to the limit of detection, which was reached at 105 m (*σ*_θ_ ~16.2). In the oxic zone (i.e., at 50 m; *σ*_θ_ ~14.9), the nitrite concentration displayed the highest values at 0.08 μmol/L (Figure 1b). It subsequently decreased but showed an additional peak (i.e., 0.04 μmol/L) in the middle of the suboxic zone (at 85 m depth; *σ*_θ_ ~16.0). In the euxinic zone, the ${\text{NO}}_{2}^{-}$ concentration was below the limit of detection. Up to 90 m depth (*σ*_θ_ ~16.0), ammonium concentrations were <0.1 μmol/L (Figure 1b). From this point onward, they gradually increased following the trend of HS^‐^, and reached ~100 μmol/L at 2,000 m depth (Figure 1b).

### 3.2 Abundance and diversity of archaeal groups in the water column

Total archaeal 16S rRNA gene copies were quantified in the water column by qPCR by using archaea‐specific primers. The abundance of total archaeal 16S rRNA gene copies/L ranged by two orders of magnitude, that is, from 0. 045 to 2.4 × 10^8^ copies/L. It was high at the oxic–suboxic interface (70–80 m; ca. 1 × 10^8^ copies/L) and revealed a maximum within the suboxic zone (105 m; 2.4 × 10^8^ copies/L) and in the deepest euxinic waters (2,000 m; 1.2 × 10^8^ copies/L) (Figure 1c).

In order to determine the archaeal diversity, partial 16S rRNA gene sequences were retrieved by amplicon sequencing of the prokaryotic DNA extracted from the SPM across the vertical profile of the Black Sea (Table 1; Figure 1d). The percentage of archaeal 16S rRNA gene reads of the total (i.e., bacterial plus archaeal 16S rRNA gene reads) ranged from 1.2% to 18.5%. The percentage of archaeal 16S rRNA gene reads was higher between 70 and 100 m depth (8%–18% of the total) and then decreased down to 1.2% of the total at 170 m. At greater depths, it slightly increased at 2,000 m to approximately 5% of the total. The distribution of the archaeal 16S rRNA gene reads (between 50 and 2,000 m) in different archaeal groups is specified in Table 1 and Figure 1d (only those archaeal groups with % 16S rRNA gene reads higher than 3% were plotted and discussed below).

**Table 1:**
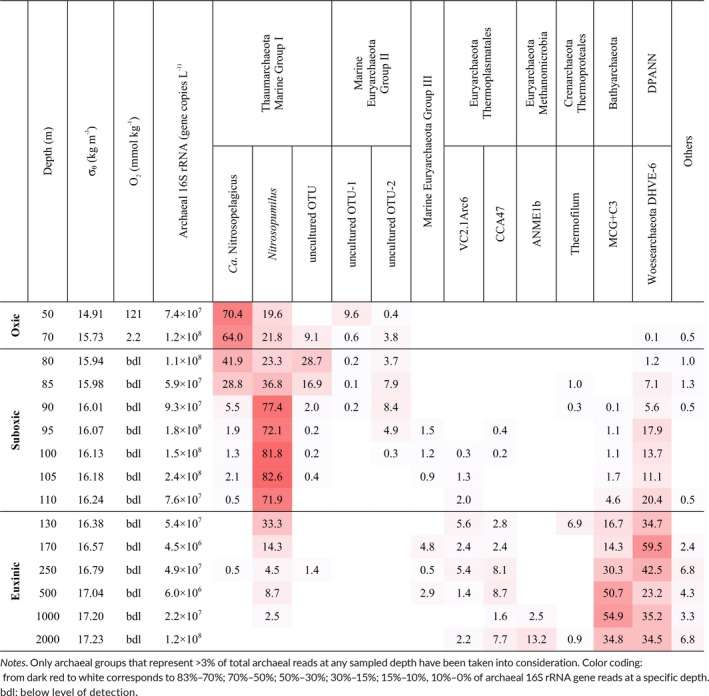
Physical properties (potential density and oxygen concentration), absolute abundance of the archaeal 16S rRNA gene copies, and the distribution of the reads over the various detected archaeal phylogenetic groups per depth in the Black Sea water column at station PHOX2

**Figure 2:**
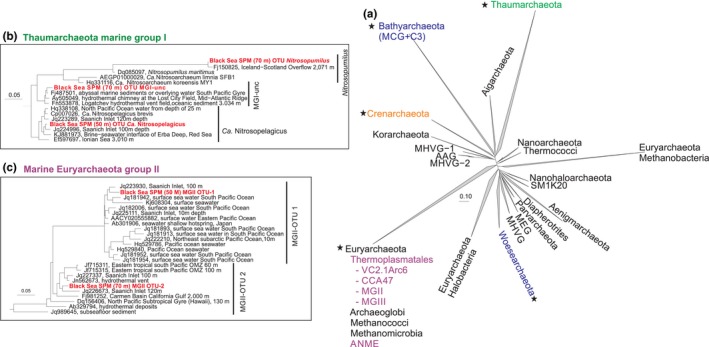
(a) General archaeal 16S rRNA gene tree revealing the phylogenetic positions of the archaeal groups (indicated with a star) detected in the Black Sea and discussed in the text. The specific subgroups and their distribution are shown in Fig. 1. (b and c) Sub‐trees showing the phylogenetic positions of the three OTUs of Thaumarchaeota, and the two MGII OTUs in relation to other relevant sequences. The trees were obtained using 16S rRNA gene sequences of the Silva release 123 and those obtained in this study. [Colour figure can be viewed at wileyonlinelibrary.com]

In the oxic and suboxic zones (up to 110 m depth), Thaumarchaeota comprised most of the archaeal reads (72%–95%). The reads attributed to the Thaumarchaeota were classified in three OTUs; one including reads closely related to the 16S rRNA gene sequence of *Candidatus* Nitrosopelagicus brevis (Santoro et al., 2015), a second one to sequences closely related to the thaumarchaeon *Nitrosopumilus maritimus* (Könneke et al., 2005), and a third group including sequence reads affiliated to an uncultured MGI cluster (see Figure 2a). The relative abundance of the sequences related to *Ca*. Nitrosopelagicus was highest at 50–70 m (64%–70% of total archaeal 16S rRNA gene reads) and then decreased (30%–42%), while the relative abundance of the sequences affiliated to the MGI uncultured OTU (named here as MGI‐unc) increased at 80–85 m (up to 30%). The 16S rRNA gene sequences affiliated to *Nitrosopumilus* were dominant in suboxic waters (72%–83% of total archaeal reads between 90–110 m; Table 1; Figure 1d). Sequences closely related to the euryarchaeotal MGII (Figure 2b) were also present above and within the suboxic zone, with 10% of the reads attributed to MGII OTU‐1 at 50 m depth, while MGII OTU‐2 comprised 5%–9% of the archaeal 16S rRNA gene reads at 70–95 m depth (Figure 1d). Both MGII OTUs were not related to any cultured relatives. Other archaeal groups such as MGIII, Thermoplasmatales, Bathyarchaeota (named here MCG for simplicity), and group C3 (a subgroup within the Bathyarchaeota) were also present in the suboxic waters but at low relative abundance (average 1.5% of the archaeal 16S rRNA gene reads; Figure 1d).

In the euxinic zone, different archaeal groups of those present in the oxic and suboxic zones comprised the majority of the archaea 16S rRNA gene reads (Table 1; Figure 1d). Archaea closely related to uncultured Thermoplasmatales, that is, falling in the VC2.1Arc6 and CCA47 groups, made up to 5%–6% reads between 130 and 250 m, and 250–2,000 m depth, respectively. The percentage of reads attributed to MCG + C3 archaea increased in the euxinic zone, reaching maximum values at 500–2,000 m (average 47%). At 2,000 m depth, 13% of the reads were attributed to the Euryarchaeota ANME‐1b group. MGIII archaea were represented by approximately 5% of the reads at 170 m depth and 3% at 500 m depth. DPANN Woesearchaeota Deep Hydrothermal Vent Group (DHVE)‐6, whose relative abundance increased already in the suboxic zone (up to 18%), continued increasing in the euxinic zone with values reaching 59% (Table 1; Figure 1d).

To further investigate the diversity of Thaumarchaeota, the archaeal *amoA* gene was studied in SPM from 50, 85, 100, 500, and 2,000 m depth. All retrieved *amoA* gene coding sequences were closely related to *amoA* gene sequences previously detected in shallow (0–200 m) marine waters (“Water column A” clade of Supporting Information Figure S3). Most (90%) sequences from 50 m grouped in cluster 1 of that clade (Supporting Information Figure S3) and are closely related to the *amoA* gene of *Ca*. Nitrosopelagicus. The majority of the *amoA* gene coding sequences obtained from 85, 100, 500, 2,000 m depth grouped in cluster 2 and are more closely related to *amoA* gene of *Nitrosopumilus* (Supporting Information Figure S3).

### 3.3 Distribution of archaeal intact polar lipids

To examine the distribution of archaeal IPLs across the Black Sea water column, 193 individual IPLs (Supporting Information Table S2) were targeted for analysis using UHPLC‐HRMS (Besseling et al., 2018). Table 2 shows the distribution of the 38 individual archaeal IPLs that were actually detected. These are reported as a heat map of the relative abundance (%) of the total amounts of each individual IPL over the full depth range. The lack of authentic standards for these IPLs makes it difficult to compare the relative abundances of all IPLs at one specific water depth. The detected IPL types included a number of different headgroups attached in various combinations to GDGT‐0 to GDGT‐4, crenarchaeol, OH‐GDGT‐0 to OH‐GDGT‐4, and archaeol as core apolar lipids (see Supporting Information Figure S1 for structures of core lipids). Detected headgroups are as follows: monohexose (MH); dihexose (DH), here indicating two MH moieties when attached to crenarchaeol or the other GDGTs, or one DH moiety when attached to archaeol; hexose phosphohexose (HPH), corresponding to two headgroups, namely one MH group and one phosphatidyl MH; methyl‐hexose phosphohexose (MeHPH); 2‐phosphatidylglycerol (2PG); monohexose 2‐phosphatidylglycerol (MH‐2PG); hexose‐glucuronic acid (HgluA); phospho‐dihexose (PDH); phosphatidylglycerol (PG); phosphatidylethanolamine (PE); phosphatidylserine (PS). Most of the targeted archaeal IPLs have been detected previously in environmental IPL surveys (Besseling et al., 2018; Liu et al., 2012; Meador et al., 2015; Rossel, Elvert, Ramette, Boetius, & Hinrichs, 2011; Rossel et al., 2008; Schubotz, Wakeham, Lipp, Fredricks, & Hinrichs, 2009). Two new IPLs, MeHPH‐GDGT‐0 and HgluA‐archaeol, however, are reported here for the first time (see Supporting Information Figures S4 and S5 for details).

**Table 2:**
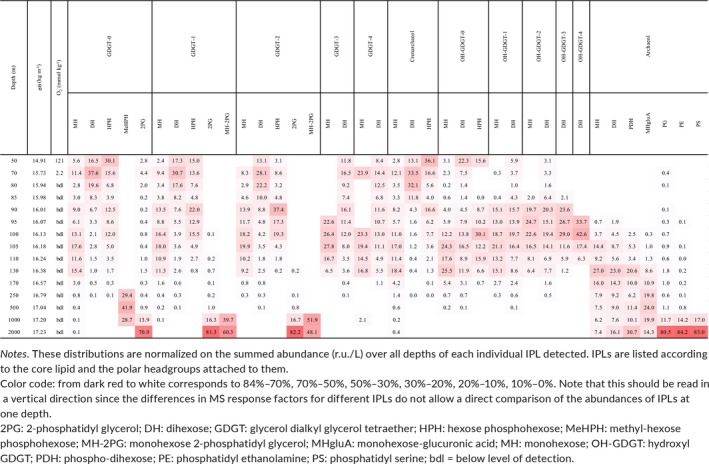
Depth distributions of the archaeal IPLs detected in the Black Sea water column

GDGT‐0 to GDGT‐2 and crenarchaeol were detected with MH, DH, and HPH as headgroups; GDGT‐3 and GDGT‐4 only occurred with MH and DH headgroups. In addition, GDGT‐0 to GDGT‐2 also occurred as 2PG IPLs, and GDGT‐1 and GDGT‐2 as MH‐2PG IPLs (Table 2). The depth profiles of these IPLs were basically independent of the core GDGT composition. For example, all MH‐GDGTs had a low relative abundance in the shallow oxic and upper suboxic waters (50–85 m; *σ*_θ_ ~14.9–15.9) but tended to increase toward the core suboxic and the upper euxinic zones (90–130 m; *σ*_θ_ ~16.0–16.4). In addition, MH‐GDGT‐4 showed high relative abundance also at 70 m. All DH‐GDGTs had higher relative abundance in the upper suboxic waters, then rapidly decreased with depth except for DH‐GDGT‐3 and DH‐GDGT‐4, whose relative abundance remained stable down to 105 m (*σ*_θ_ ~16.2). The HPH‐GDGTs displayed a maximum in relative abundance in the oxic zone (50 m; *σ*_θ_ ~14.9) and again at 90 m (*σ*_θ_ ~16.0) in the core suboxic zone. HPH‐GDGT‐2 was an exception as it only had a maximum at 90 m depth (Table 2). The 2PG‐GDGT‐0 to 2PG‐GDGT‐2 and the MH‐2PG‐GDGT‐1 and MH‐2PG‐GDGT‐2 were only detected in the deep euxinic waters (1,000–2,000 m; *σ*_θ_ ~17.2) and had their highest relative abundance at 2,000 m. In addition, MeHPH‐GDGT‐0, one of the novel IPLs (Supporting Information Figure S4), had a profile that differs from all other IPL‐GDGTs as it was only detected at a discrete depth interval within the deep euxinic water column (250–1,000 m; *σ*_θ_ ~16.8–17.2; Table 2).

A distinct other group of archaeal IPLs contained OH‐GDGT‐0 to OH‐GDGT‐4 as CLs (Table 2). They primarily had MH and DH as polar headgroups for OH‐GDGT‐1 and OH‐GDGT‐2, and just DH for OH‐GDGT‐3 and OH‐GDGT‐4. OH‐GDGT‐0 was also present as an HPH IPL. In general, these IPLs occurred between 90 and 130 m (*σ*_θ_ ~16.0–16.4), that is, in the lower suboxic and upper euxinic waters. The DH‐OH‐GDGT‐1 and DH‐OH‐GDGT‐2 were also present, albeit at lower relative abundance, in shallower waters. In contrast, the relative abundance of DH‐ and HPH‐OH‐GDGT‐0 also peaked in the oxic surface waters at 50 m, in addition to a peak in the core suboxic zone (i.e., at ca. 100–105 m; Table 2).

The archaeol‐based IPLs showed an entirely different distribution than the GDGT‐based IPLs. They were undetectable or present only in traces down to ~100 m, where they started to increase and displayed their maxima below the HS^‐^ redoxcline and in the euxinic zone. The archaeol IPLs showed depth distributions that strongly varied depending on the polar headgroups. The relative abundance of MH/DH/PDH‐archaeol increased gradually from ca. 95 to 100 m toward 130 m, where they reached a maximum. DH/PDH‐archaeol showed a second maximum at 2,000 m (Table 2). The novel HgluA‐archaeol (see Supporting Information Figure S5 for details) was present only in traces down to 130 m where its relative abundance started to increase and had a maximum at 250–1,000 m (Table 2). The PG/PE/PS‐archaeol IPLs were mainly detected between 1,000 and 2,000 m and displayed a prominent maximum at 2,000 m (Table 2).

### 3.4 Statistical analyses

**Figure 3:**
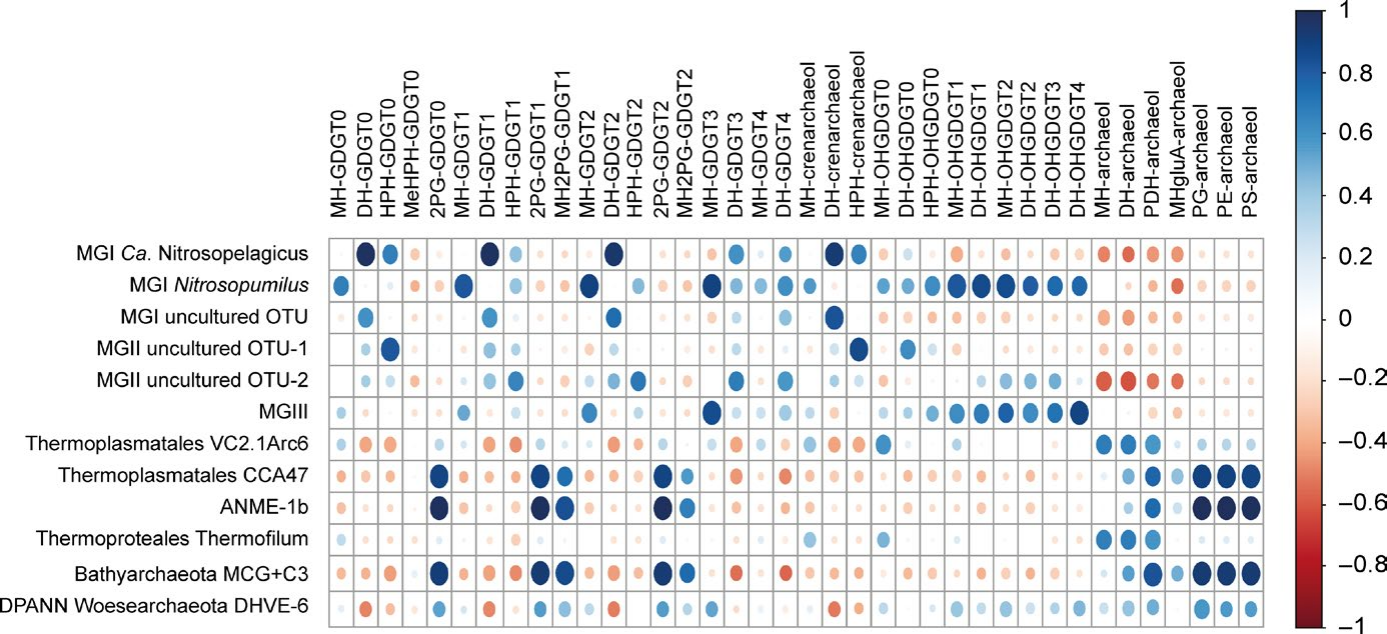
Dot plot of the correlation matrix obtained by applying a Pearson analysis to the total archaeal 16S rRNA gene reads (copies/L) of the archaeal groups and to the absolute abundances of the archaeal intact polar lipids (IPLs) (response units per Liter; r.u./L) detected in the Black Sea water column at station PHOX2. The size of the dot and the intensity of its color relate to the degree of correlation. Dark blue corresponds to *r* values of +1, indicating a strong positive linear correlation between the concentration of the archaeal IPL and archaeal gene reads; white corresponds to *r* values of 0, indicating that no correlation exists; dark red corresponds to *r* values of −1, indicating a strong negative linear correlation. [Colour figure can be viewed at wileyonlinelibrary.com]

Three correlation matrices were obtained with the available dataset. The first was developed to corroborate or dismiss the tentative assignment of specific IPLs to specific archaeal groups. To this end, the absolute abundance of specific archaeal groups was estimated from the total archaeal 16S rRNA gene concentration (copies/L; Supporting Information Table S3) obtained by qPCR and the relative abundance of the different archaeal groups as encountered by amplicon sequencing (Table 1). Potential biases in this approach are a varying copy number (we assumed one 16S rRNA gene copy number per genome), a similar amplification of 16S rRNA genes, potential differences in lysis of cells, and the use of different primers for qPCR and amplicon sequencing. The “semi‐quantified” absolute abundance of specific archaeal groups was correlated with the absolute abundances of the archaeal IPLs expressed as response units per Liter (r.u./L; Supporting Information Table S4) as detected with UHPLC‐HRMS for each depth (Figure 3; Supporting Information Table S5). It reveals clear positive correlations between various archaeal IPLs and archaeal groups in the water column of the Black Sea, suggesting potential relationships. In a second analysis, the absolute abundance of the various archaeal groups detected at each depth was correlated with each other (Supporting Information Figure S6; Supporting Information Table S6). This was meant to identify spurious correlations between specific archaeal IPLs and archaeal groups provided by the first correlation analysis, which are simply due to different archaeal groups occupying the same niche. This was apparent for MGI *Ca*. Nitrosopelagicus, MGI‐unc OTU, and MGII OTU‐1, for MGI *Nitrosopumilus*, MGIII and DPANN Woesearchaeota, and for Thermoplasmatales CCA47, euryarchaeotal ANME‐1b, Bathyarchaeota (MCG + C3). The abundance of the DPANN Woesearchaeota showed weaker positive correlations with quite some groups of archaea residing in the deeper euxinic waters. The fact that some of these archaea occupy the same niches complicates the assignment of sources of archaeal IPLs as will be discussed.

A third correlation analysis (Supporting Information Figure S7; Supporting Information Table S7) was performed on the depth profiles of archaeal IPLs quantified in response units per Liter (r.u./L; Supporting Information Table S4) to reveal potential similarities and hence potential similar sources and will be discussed where appropriate.

## 4 DISCUSSION

We have investigated the distribution of different groups of archaea in the Black Sea water column by means of a 16S rRNA amplicon sequencing approach. This revealed that the number of different archaeal groups detected was substantially higher in comparison with previous studies of the Black Sea water column (cf. Coolen et al., 2007; Lin et al., 2006; Vetriani et al., 2003; Wakeham et al., 2007). Our data allow associating certain archaeal groups with specific niches within the water column. In addition, we have encountered a substantial variation in the distribution of archaeal IPLs and, by using a novel comprehensive approach for comparison with the gene‐based data, we discuss potential specific sources associated to specific IPL archaeal lipids detected in the water column (Table 2).

The total archaeal abundance (based on archaeal 16S rRNA gene quantification) in the Black Sea water column at the time of sampling reached highest numbers in both the suboxic and in the bottom euxinic waters (Figure 1c). A peak abundance of archaea at 90–110 m depth has previously been reported in the central Black Sea by fluorescence in situ hybridization (Wakeham et al., 2007), but the second maximum has remained unnoticed, perhaps because of the under sampling of deep waters of the Black Sea in previous studies. The higher archaeal diversity we found throughout the water column, compared to earlier studies, is due to the improved resolution of the more advanced 16S rRNA gene amplicon sequencing technique used here. The archaeal groups detected include various Thaumarchaeota, Euryarchaeota of the MGII and MGIII, Thermoplasmatales, ANME‐1b, Woesearchaeota, and MCG (Table 1; Figures 1d and 2).

### 4.1 Ammonia‐oxidizing Thaumarchaeota reside predominantly in oxic and suboxic waters

In the oxic and suboxic waters, members of the Thaumarchaeota phylum were dominant representing 86%–99.5% of all archaeal sequences detected with the amplicon sequencing approach (Table 1). Thaumarchaeota closely related to *Ca*. Nitrosopelagicus were the dominant archaeal group in the fully oxygenated surface waters, where they represented 67%–78% of all Thaumarchaeota, and became minor (<7%) at >85 m. This is supported by the *amoA* diversity revealed by the protein‐coded sequence analysis since in the shallow oxic waters most of the *amoA* sequences were closely related to that of *Ca*. Nitrosopelagicus (Supporting Information Figure S3). The genome of the thaumarchaeon *Ca*. Nitrosopelagicus, which was enriched from open ocean water, codes for proteins involved in stress responses upon UV radiation and reactive oxygen species, suggesting adaptations to the oligotrophic surface ocean (Santoro et al., 2015). The preference for the upper water column of *Ca*. Nitrosopelagicus agrees with its dominant presence in the Black Sea uppermost water column. The reasons for this niche preference, for example, metabolic advantage, competition with other microbial groups, remain unresolved. However, the low ammonia concentrations in waters from 50 to 85 m depth (i.e., on average 0.06 μM; Figure 1b) are compatible with the oligotrophic lifestyle predicted for the nitrifier *Ca*. Nitrosopelagicus. In addition, sequences closely affiliated to the MGI‐unc OTU (with no closely related cultured relatives) were also detected in the oxic/upper suboxic waters but peaked in absolute abundance at 80 m, where it contributed 31% of all Thaumarchaeotal 16S rRNA gene reads. In the basal part of the suboxic zone (90–110 m depth), the summed relative abundance of these two archaeal OTUs decreased to <10% of the total Thaumarchaeotal 16S rRNA gene reads and they were dominated by *Nitrosopumilus*‐related sequences, which represented up to 83% of the total archaeal 16S rRNA gene reads (Table 1). Again, this is supported by the nucleotide‐derived amino acid analysis of *amoA* as most of the *amoA* sequences in the suboxic waters were closely related to that of *Nitrosopumilus* (Supporting Information Figure S3). *Nitrosopumilus* has previously been reported to thrive at the redoxclines of the Black Sea (Coolen et al., 2007; Lam et al., 2007) and of the Gotland Deep in the Baltic Sea (Labrenz et al., 2010). In the study of Coolen et al. (2007), the MGI 16S rRNA gene diversity was evaluated by denaturing gradient gel electrophoresis, and also three different MGI OTUs where detected throughout the water column of three stations in the Black Sea, specifically sequences closely related to *N. maritimus* were detected in oxic/suboxic waters. The dominance of *Nitrosopumilus* 16S rRNA gene sequences in the suboxic zone in our study coincided with the maximum of total archaeal 16S rRNA gene copies (i.e., at 105 m; Figure 1c,d). This has been attributed to the preference of Thaumarchaeota for niches with relatively low concentrations of oxygen and ammonia, enabling this group to outcompete bacterial nitrifiers (see Erguder, Boon, Wittebolle, Marzorati, & Verstraete, 2009 for a review). From 90 to 110 m, sulfide concentrations are apparently still low enough (<5 μM) to allow the proliferation of Thaumarchaeota. These archaea are believed to be inhibited by higher sulfide concentrations, but also have an advantage over bacteria in sulfide‐containing waters because the sulfide inhibition is more severe for bacterial than for archaeal nitrifiers (Erguder et al., 2009). It is also possible that the interaction of *Nitrosopumilus* with sulfur‐oxidizing bacteria (SOB) in the upper redoxcline of the Black Sea allows the proliferation of this archaeal group. In fact, a previous study has reported the successful establishment of co‐cultures between sedimentary ammonia‐oxidizing archaea (AOA) and SOB (Park et al., 2014). We hypothesize that this may explain the co‐existence of these groups in environments characterized by a redox gradient. Indeed, previous studies have reported the presence of SOB at the Black Sea redoxcline, that is, the uncultured group SUP05 and members of the Epsilonproteobacteria (Glaubitz, Kießlich, Meeske, Labrenz, & Jürgens, 2013; Grote, Jost, Labrenz, Herndl, & Jürgens, 2008), which may support the niche preference of *Nitrosopumilus*‐related Thaumarchaeota at the redoxcline of the Black Sea. In the euxinic zone, thaumarchaeotal 16S rRNA gene reads related to *Nitrosopumilus* were also detected although their contribution to the total archaeal reads readily declined with increasing depth (Figure 1d). The conditions in the shallow euxinic zone (absence of oxygen and increasing sulfide concentrations) are believed to be incompatible with the physiology of the cultivated members of the Thaumarchaeota. This together with the fact that the 16S rRNA gene sequences recovered from these euxinic waters were closely related to the 16S rRNA gene sequences of the *Nitrosopumilus* group of the suboxic waters supports the hypothesis that this DNA signal is preserved and exported from the chemocline rather than derived from living thaumarchaeotal cells.

Considering the gene‐based results, Thaumarchaeota are likely the predominant source for the archaeal IPLs present in the waters of the oxic and suboxic zones and the changes in the thaumarchaeotal composition are probably the most important factor for the observed changes in IPL distribution (Tables 1 and 2; Figure 1d). Culture studies have shown that Thaumarchaeota synthesize mainly GDGT‐0 and crenarchaeol and, in lower abundance, GDGT‐1 to GDGT‐4 as their core lipids (Elling et al., 2014, 2017; Pitcher, Villanueva et al., 2011; Schouten et al., 2008; Sinninghe Damsté et al., 2002, 2012). More recently, mono‐ (OH‐) and dihydroxy‐ (2OH‐) GDGTs have also been reported in thaumarchaeotal cultures (Liu et al., 2012). This is in good agreement with our data since we detected predominantly crenarchaeol and GDGT‐0 to GDGT‐4 as well as OH‐GDGTs, mostly with MH, DH, and HPH head groups, in the oxic/suboxic zones (Table 2). The IPL‐crenarchaeol absolute abundances (Supporting Information Table S4) were highest in the oxic zone confirming Thaumarchaeota as its main biological source since 90%–95% of 16S rRNA gene reads belongs to this group (Table 1; Figure 1c). Assuming similar response factors, HPH‐crenarchaeol was the prevailing IPL at 50 m depth, but at 70–80 m DH‐crenarchaeol was predominant (Table 2). The Pearson correlation analysis showed that both DH and HPH‐crenarchaeol were positively correlated with the presence of the MGI *Ca*. Nitrosopelagicus and the MGI‐unc OTU (Figure 3). MGII OTU‐1 also correlated with DH and HPH‐crenarchaeol, but this is likely due to the fact that they occupy the same niche as is evident from the correlation matrix (Supporting Information Figure S6).

The high percentage of 16S rRNA gene reads attributed to the *Nitrosopumilus* OTU and the maximum archaeal 16S rRNA gene abundance detected in the suboxic water (Table 1; Figure 1c,d) coincided with high relative abundances of OH‐GDGT IPLs, with MH, DH, and HPH as polar headgroups (up to the order of 10^7^ r.u./L; Table 2). This suggests that *Nitrosopumilus*‐like Thaumarchaeota might be the source of the OH‐GDGT IPLs, which is corroborated by the positive scores obtained in the correlation matrix (Figure 3 and Supporting Information Table S6). Indeed, the relative abundance of OH‐GDGT‐1 and OH‐GDGT‐2 with MH and DH as polar headgroups and, to a certain degree, of DH‐OH‐GDGT‐3 and DH‐OH‐GDGT‐4 was higher in the suboxic waters compared to the shallower oxic and the deeper euxinic waters (Table 2). Previous studies reporting glycosidic OH‐GDGTs in marine sediments (Lipp & Hinrichs, 2009) and cultures (Liu et al., 2012) tentatively proposed them as originating from both crenarchaeota and euryarchaeota.

At a sampling station located at the center of the basin with comparable conditions to the one reported in this study, Schubotz et al. (2009) reported MH‐GDGT‐0 and MH‐GDGT‐crenarchaeol as the main archaeal IPLs in the suboxic waters, accounting together for up to 80% in relative abundance, but they did not detect HPH‐crenarchaeol. MH IPLs, whose glycosidic polar headgroup is generally considered as deriving from preservation rather than living biomass (Schouten et al., 2012), may be attributed to a Thaumarchaeota fossil signal sinking down from the chemocline. Schubotz et al. (2009) also reported GDGT‐2 and GDGT‐3 attached to DH and to an unidentified headgroup MH341—tentatively identified as a DH‐OH‐GDGT in a subsequent study (Liu et al., 2012)—as secondary IPLs and suggested methanotrophic euryarchaeota as a possible source (Schubotz et al., 2009). However, in our study, the dominant presence of *Nitrosopumilus* over the other archaeal groups in the suboxic zone and the highly positive scores in the correlation analysis strongly suggests this group as the most likely source of the DH‐OH‐GDGT IPLs (Figures 1c and 3).

### 4.2 MGII Euryarchaeota in the suboxic zone

Sequences of the MGII OTU‐1 (Figure 2) were almost exclusively detected in the oxic waters at 50 m depth, representing ca. 10% of the archaeal 16S rRNA gene reads (Table 1; Figure 1d). Below 50 m, calculated cell numbers (Supporting Information Table S3) declined by 1–2 orders of magnitude, and at depths >90 m, this group was no longer detected. This indicates a preference of this MGII group for the photic zone, and it may be possible that they were even more abundant in the shallower waters (<50 m) not examined in this study. The presence of genes encoding proteorhodopsin homologs in metagenomes of MGII archaea suggested a photoheterotrophic lifestyle (Iverson et al., 2012), which is in agreement with their niche in the Black Sea.

MGII OTU‐2 occupied a different niche than MGII OTU‐1 with calculated copy numbers maximizing at 90–95 m in the suboxic zone (Supporting Information Table S3), suggesting an adaptation to very low oxygen concentrations. The negative correlation of the abundances of these two uncultured MGII OTUs (Supporting Information Figure S6; Supporting Information Table S6) is a confirmation of the genetic diversity occurring between these OTUs. Because of the abundance of the Thaumarchaeota, MGII OTU‐2 reads never exceeds 9% (Table 1; Figure 1d). Although early studies indicated the predominance of MGII archaea in surface waters of the ocean (Massana, Murray, Preston, & DeLong, 1997; Massana et al., 2000), subsequent studies revealed that other ecotypes exist in deeper waters (see Zhang et al., 2015 for a review). Genomic analyses of these deep water MGII suggested the capacity of anaerobic respiration using organic matter as electron donor (Orsi et al., 2015), which would be in good agreement with the niche observed in Black Sea waters. The absence of MGII OTU‐2 in the basal part of the suboxic zone (i.e., they are absent in waters below 105 m) suggests that these archaea are sensitive to low concentrations of sulfide.

As mentioned above, the lipid composition of the upper suboxic waters (80–90 m depth) was dominated by GDGT‐0, GDGT‐1, and GDGT‐2 as well as crenarchaeol mostly with DH polar headgroup (Table 2), which are attributed to the MGI Thaumarchaeota. Recently, Lincoln et al. (2014a) and Wang, Wei, Wang, Hong, and Zhang (2015) provided evidence that MGII may be synthesizing GDGTs, including crenarchaeol, in the marine water column. However, this is still a highly debated topic (Lincoln et al., 2014b; Schouten et al., 2014). The question if MGII archaea are able to synthesize GDGTs, and specifically crenarchaeol, can only be ultimately answered by the cultivation of this group of archaea. In our study, in the correlation matrix of the IPLs *vs* the archaeal groups, the two MGII OTUs scored positively with several IPL‐GDGTs and especially MGII OTU‐1 was positively correlated with HPH‐GDGT‐0 and HPH‐GDGT‐crenarchaeol (Figure 3; Supporting Information Table S5). However, the correlation matrix of the archaeal groups (Supporting Information Figure S6; Supporting Information Table S6) reveals that this correlation is probably the result of MGII OTU‐1 sharing the same ecological niche with *Ca*. Nitrosopelagicus and MGI‐unc OTU (Figure 1d). This, together with the low relative abundance of MGII archaea (up to 10% of the total archaeal population) in the suboxic waters, does not allow correlating their presence with the IPLs detected here and, hence, cannot contribute in determining the IPLs synthesized by MGII archaea.

### 4.3 Other archaeal groups in the suboxic zone

Other archaeal classes, that is, the MGIII, MCG, and the DPANN Woesearchaeota, were also detected in the suboxic waters (Table 1; Figure 1d). The low relative abundance of MGIII and MCG in the suboxic waters (ca. 1% of the total archaeal 16S rRNA gene reads)rules them out as major contributors to the main IPLs (i.e., HPH‐GDGT‐1, HPH‐GDGT‐2 and DH‐OH‐GDGT‐1, DH‐OH‐GDGT‐2) in the suboxic water in spite of the positive correlation, the two archaeal groups had with those IPLs (Figure 3; Supporting Information Table S5). DPANN Woesearchaeota contributed to an average of 12% of the total archaeal 16S rRNA gene reads in the suboxic waters (Table 1; Figure 1d), which is relatively minor compared to the percentage of the *Nitrosopumilus*‐like sequences. In addition, recent studies suggest that members of the DPANN Woesearchaeota have only small genomes lacking the genes required for the lipid membrane biosynthesis (Castelle et al., 2015; Villanueva, Schouten, & Sinninghe Damsté, 2017). If so, they would be expected to depend on recycling extracellular lipids to build their membranes and therefore do not synthesize their own lipids, but contribute to the total IPL as recently suggested by Lipsewers, Hopmans, Sinninghe Damsté, and Villanueva (2018).

### 4.4 Anaerobic methane‐oxidizing archaea reside in deep sulfidic waters

The 16S rRNA gene amplicon sequencing analysis indicated that Euryarchaeota Methanomicrobia ANME of the subgroup 1b were the only ANME representatives in the Black Sea euxinic waters. They were only detectable at 1,000 m depth (2.4%) and reached 13% of the total archaeal reads at 2,000 m depth (Table 1; Figure 1d). Considering the quantification of total archaeal 16S rRNA gene, which peaks at 2,000 m (Figure 1c) and the high percentage of 16S rRNA gene sequences attributed to ANME‐1b, the group was a significant component of the archaeal community in the deep euxinic waters of the Black Sea at the time of sampling (Figure 1d). Members of the ANME‐1 have previously been detected in Black Sea carbonate chimneys (Blumenberg et al., 2004). Genetic evidence has also indicated the presence of a community dominated by ANME‐1 in the deep Black Sea, although they represented <2% of the total archaeal copies at the deepest water mass (i.e., 1,500 m) analyzed (Schubert et al., 2006). Wakeham et al. (2007) employed specifically designed ANME‐1 FISH probes but were not able to detect ANME‐1. Schubert et al. (2006) also reported the presence of ANME‐2 in the shallower waters of the euxinic zone, but this is not confirmed by our data.

Previous environmental studies have suggested that ANME‐1 membrane lipids include as core lipids mostly GDGT‐0 to GDGT‐2 and, in much lower abundance, archaeol (Blumenberg et al., 2004; Pancost et al., 2001; Wakeham et al., 2003). In euxinic Black Sea waters, the ANME‐1 distribution was characterized by approximately equal amounts of GDGT‐0, GDGT‐1, and GDGT‐2 (Wakeham et al., 2003). The stable carbon isotopic composition of the biphytanes comprising these GDGTs revealed that they were depleted in ^13^C, establishing a direct link with methane in the water column (Wakeham et al., 2003). ANME‐2, the other major methanotrophic archaeal subgroup, predominantly produces *sn*‐2‐OH‐archaeol as a CL (Blumenberg et al., 2004; Rossel et al., 2008, 2011; Stadnitskaia et al., 2005; Wakeham et al., 2007). The profiles of the 16S rRNA gene ANME‐1b reads and of 2PG‐GDGT‐0, 2PG‐GDGT‐1, and 2PG‐GDGT‐2 (Tables 1 and 2), all increasing substantially at 1,000–2,000 m depth (Figure 3; Table 2 and Supporting Information Table S6), revealed a significant positive correlation (*r* > 0.99). This strongly suggests that these IPLs are synthesized by ANME‐1b in the deepest part of the euxinic zone. In good agreement with the characteristic CL distribution of ANME‐1 in deep Black Sea waters (Wakeham et al., 2003, 2007), these three 2PG IPLs have approximately the same concentration (Supporting Information Table S4). These IPLs were previously attributed to communities dominated by ANME‐1 associated with sulfate‐reducing bacteria of the *Desulfosarcina‐Desulfococcus* (DSS) branch in methane‐rich sediments and microbial mats of hydrocarbon seeps from multiple locations including the Black Sea floor (Meador et al., 2015; Rossel et al., 2011). The MH‐2PG‐GDGT‐1 and MH‐2PG‐GDGT‐2, which showed the same depth distribution and also have positive correlation scores with the ANME‐1b group (*r* = 0.84 and 0.68), are likely also synthesized by this archaeal group (Table 2 and Supporting Information Table S5), as previously suggested by Rossel et al. (2011). Remarkably, however, we detected the characteristic 2PG IPLs already at shallower depths in the euxinic zone, albeit at concentrations that are 2–3 orders of magnitude lower. This suggests that ANME‐1 also occurs at shallower depths, in agreement with earlier reported CL GDGT distributions (Wakeham et al., 2003, 2007). This suggests that IPL analysis in some cases is more sensitive than 16S rRNA gene amplicon sequencing analysis to detect the presence of these methane‐oxidizing archaea.

PS‐, PG‐, and PE‐archaeol also displayed depth profiles comparable to the three 2PG‐GDGTs in the deep euxinic waters (Table 2) and also showed a highly significant correlation (*r* > 0.99) with the depth profile of the ANME‐1 reads (Figure 3; Supporting Information Table S6). PS‐ and PG‐archaeol have previously been tentatively assigned to ANME‐2 and ANME‐3 (Rossel et al., 2008, 2011), archaeal groups that were not revealed to be present in the Black Sea water column by our 16S rRNA gene amplicon sequencing approach. PE‐archaeol was not detected by these authors, although it has been reported in marine sediments at several locations (Schubotz, Lipp, Elvert, & Hinrichs, 2011; Yoshinaga et al., 2011) and in cultures of the hyperthermophilic archaeon *Thermococcus kodakarensis* (Meador et al., 2014). However, Wegener et al. (2016) detected PG‐archaeol in their ANME‐1 enrichment culture, in good agreement with our observations although these indicate that ANME‐1 also produces PS‐, and PE‐archaeol. Hence, our data together with previous isotope and core lipid data (Wakeham et al., 2003, 2007) indicate that ANME‐1 is a relevant contributor to the archaeal community of the deep Black Sea. This is in contrast with a previous study of IPLs in Black Sea waters by Schubotz et al. (2009) who did not detect any IPLs that could be linked to ANME archaea.

### 4.5 Other archaea thriving in the euxinic zone

In addition to ANME‐1b, members of the Thermoplasmatales VC2.1Arc6 and CCA47, Crenarchaeota of the Thermoproteales Thermofilum, Bathyarchaeota MCG, and MGIII were predominantly detected in the euxinic zone (Table 1; Figure 1d), suggesting that all these archaea are anaerobic and can deal with high sulfide concentrations. DPANN Woesearchaeota DHVE‐6 also occurred in the suboxic zone, but their relative abundance increased substantially with depth (average 37% from 130 to 2,000 m depth), reaching a maximum of almost 50% of archaeal reads at 170 m depth. Bathyarchaeota represented the most prominent group below 170 m. They were barely detectable (i.e., <1% of archaeal reads) in the deeper suboxic zone (~105–110 m), then reached >10% at 130 m and reached the highest levels (55%) in the deeper part of the euxinic zone (1,000 m depth). Other less abundant groups include two subgroups of the Thermoplasmatales (average of 9%), which occurred throughout the euxinic zone with no clear maximum, and Thermoproteales Thermofilum, which only appeared at 130 m at a substantial relative abundance (i.e., 7%; Table 1; Figure 1d). The 16S rRNA gene sequences affiliated to the Thermoplasmatales and Bathyarchaeota have been mostly detected in sediments (e.g., Lloyd et al., 2013). Thermoplasmatales cluster VC2.1Arc6 was originally described in deep‐sea hydrothermal vents on the Mid‐Atlantic Ridge (Reysenbach & Longnecker, 2000), but these archaea have also been detected in deep‐sea sediments of other locations such as the Marmara Sea (Quaiser, Zivanovic, Moreira, & López‐García, 2011). The Thermoplasmatales CCA47 group was originally identified in oxygen‐depleted marine environment and anoxic subsaline sediments (Ferrer et al., 2011; Stoeck & Epstein, 2003), which is compatible with the conditions found in the Black Sea water column. All members of the complex archaeal community thriving in the euxinic water column detected in our study (Table 1; Figure 1d) have remained uncultivated. Despite this, we can infer they use some metabolic strategy, either independently or requiring some sort of cooperation with bacterial lineages, to deal with the increasing concentrations of sulfide found. The high abundance of MCG archaea in the euxinic zone is remarkable as it is an archaeal phylum typically associated with marine subseafloor sediments (Biddle et al., 2006; Durbin & Teske, 2011; Fry, Parkes, Cragg, Weightman, & Webster, 2008; Inagaki et al., 2003; Kubo et al., 2012). However, these archaea have also been reported to be present in karstic lakes with high sulfide concentrations (i.e., 150 μM; Fillol, Alexandre Sànchez‐Melsió, & Borrego, 2015), which could indicate a role of MCGs in sulfide‐rich pelagic systems. Another remarkable finding of our study is the detection of DPANN Woesearchaeota DHVE‐6, which comprised on average of 40% of the total archaeal 16S rRNA gene reads in the euxinic waters, representing ca. 1 × 10^7^ cells/L. This subgroup of the DPANN superphylum is still enigmatic and recent reports based on metagenomic sequencing indicate that they might be involved in carbon and hydrogen metabolism, probably associated with symbiotic and/or fermentation‐based lifestyles (Castelle et al., 2015), which is compatible with their presence in anoxic waters. It is plausible that they are also involved in the sulfur cycle in view of their high abundance in the euxinic waters of the Black Sea, but future studies would need to address this possibility.

By comparing our lipidomic and genetic datasets, we may provide clues as to which membrane lipids the uncultivated archaeal groups in the euxinic waters of the Black Sea produces. The high abundance of the DPANN archaea (Table 1) would make them an important IPL source in these waters, but their apparent lack of lipid membrane biosynthetic potential (Castelle et al., 2015; Lipsewers et al., 2018; Villanueva et al., 2017) makes this implausible. A further complication in this approach is that the abundances of almost all dominant archaeal groups in the euxinic zone correlate positively with each other (Supporting Information Figure S6). The correlations between the abundance of ANME‐1b, Thermoplasmatales CCA47, and Bathyarchaeota MCG + C3 were high (*r* = >0.91; Supporting Information Table S6), and the correlations of those groups with Thermoplasmatales VC2.1Arc6, Thermoproteales, and DPANN were moderately positive (Supporting Information Figure S6; Supporting Information Table S6). In contrast, the abundance of MGIII, whose distribution spanned throughout the suboxic and the upper euxinic zones (Table 1), correlated positively only with that of the DPANN group (Supporting Information Figure S6; Supporting Information Table S6).

The composition of the IPLs present in the euxinic zone was more diverse compared to the oxic and suboxic zones, including both GDGT‐ and archaeol‐based IPLs. The dominant IPLs are based on common CLs such as GDGT‐0 to GDGT‐2 and archaeol, but attached to a broad variety of polar headgroups. Specifically, we detected 2PG attached to GDGT‐0 to GDGT‐2, MH‐2PG‐GDGT‐1 and MH‐2PG‐GDGT‐2, MH/DH/PDH/PG/PE/PS‐archaeol, and the two novel IPLs MeHPH‐GDGT‐0 and HgluA‐archaeol. Of these IPLs, the abundances of 2PG‐GDGT‐0, 2PG‐GDGT‐1, and 2PG‐GDGT‐2, and archaeol with various head groups (i.e., MH, DH, PDH, HgluA, PG, and PE) gradually increased with depth (Table 2). The concentration profiles of 2PG‐GDGT‐0, 2PG‐GDGT‐1, and 2PG‐GDGT‐2 and PG‐, PE‐, and PS‐archaeol correlated positively with each other (Supporting Information Figure S7; Supporting Information Table S7) since, as suggested earlier, they are likely produced by ANME‐1b.

Bathyarchaeota represented one of the most abundant groups in the euxinic zone (Table 1; Figure 1d), which would make this group a potential source for the most abundant IPL classes detected in the euxinic waters (Table 2). A previous study of anoxic estuarine sediments (Meador et al., 2015) proposed BDGT‐IPLs as putative biomarkers of Bathyarchaeota MCG. However, BDGTs have recently been associated to methanogens of the Methanomassiliicoccales order, which is closely related to Thermoplasmatales and euryarchaeotal DHVE‐2 (Becker et al., 2016). However, BDGT‐IPLs were not detected by this study. Although the depth profiles of the Bathyarchaeota abundance correlated significantly with those of 2PG‐GDGT‐0, 2PG‐GDGT‐1, and 2PG‐GDGT‐2 and PG‐, PE‐, and PS‐archaeol (Figure 3; *r* = 0.92–0.93, Supporting Information Table S5), these correlations were less significant than those with the ANME‐1b (see earlier). Furthermore, Bathyarchaeota already appear in the suboxic zone (ca. 90 m) and their relative abundance gradually increased with increasing depth up to the deep euxinic zone (Table 1). The ANME‐1b group and the IPLs assigned to them instead appeared only at the lowermost part of the euxinic zone (1,000–2,000 m) (Tables 1 and 2). It is interesting to note that just like the relative abundance of the Bathyarchaeota (Table 1) the IPL HgluA‐archaeol had its maximum abundance in the 250–1,000 m depth range (Table 2). At this depth, this IPL was one to two orders of magnitude higher as measured in response units compared to other IPLs (Supporting Information Table S4). Although the response factors for IPLs vary, this strongly suggests that HgluA‐archaeol is one of the dominant IPLs in the upper part of the euxinic zone and should be sourced by one of the dominant groups of archaea residing here (i.e., Bathyarchaeota). Indeed, in the correlation analysis (Figure 3), Bathyarchaeota MCG was the archaeal group that correlated most positively with HgluA‐archaeol (*r* = 0.51), although the correlation was less significant than observed for the ANME‐1 IPLs. The profile of PDH‐archaeol also showed the highest correlation with the Bathyarchaeota profile (*r* = 0.84), suggesting this IPL may also be produced by these archaea.

Two different groups of Thermoplasmatales were detected in the euxinic water column with distinct profiles (Table 1). The CCA47 group had its highest relative abundance in the deep euxinic waters (Table 1). Its abundance profile reveals a high correlation with those of ANME‐1b and Bathyarchaeota groups, which, in combination with CCA47 lower relative abundance masks potential relationships with IPLs. The other Thermoplasmatales group, VC2.1Arc6, had a more distinct distribution (Table 1). Its depth profile shows a fairly high correlation with those of MH‐ and DH‐archaeol (*r* = 0.68; Supporting Information Table S5), and these two IPL profiles are also significantly correlated with each other (*r* = 0.91; Supporting Information Table S6). This suggests that the VC2.1Arc6 archaea may be producing these two structurally related IPLs. However, the profile of the Thermofilum group, although this group was less prominent (Table 1), shows a similar correlation with the profiles of MH‐ and DH‐archaeol (*r* = 0.66–0.68; Supporting Information Table S5).

Interestingly, the newly described MeHPH‐GDGT‐0 IPL displayed a specific depth profile, being only detected in the 250 to 1,000‐ m interval (Table 2). For this, we would have expected to find a clear correspondence of this distinctive IPL profile with a specific archaeal group (Tables 1 and 2). However, there was no such clear match and the correlation analysis confirmed that none of the archaeal lineages detected by this study strongly correlated with the MeHPH‐GDGT‐0 (Figure 3; Supporting Information Table S5), suggesting an as yet unidentified source.

## 5 CONCLUSIONS

The comprehensive combination of 16S rRNA gene amplicon sequencing and lipidomic methods applied here for the first time has advanced our understanding of the diversity, distribution, and abundance of archaeal groups in the Black Sea water column. The number of archaeal groups, determined by 16S rRNA gene amplicon sequencing, and variety of archaeal lipids as determined by UHPLC‐HRMS, was higher than previously described. We observed dominance of Thaumarchaeota *Ca*. Nitrosopelagicus in the oxic and upper suboxic waters of the Black Sea water column, coinciding with a higher dominance of HPH‐crenarchaeol, while suboxic waters were dominated by the thaumarchaeon *Nitrosopumilus* and characterized by a high relative abundance of DH‐OH‐GDGT‐1/‐2, and HPH‐GDGT‐0/‐2. The euxinic waters were characterized by a broad range of archaea. Members of the Bathyarchaeota (MCG) dominated, but others such as DPANN Woesearchaeota, Thermoplasmatales, MGIII, and ANME‐1 archaea were also present. Correlation analyses support a connection between the IPLs MH‐2PG‐GDGT‐1 and MH‐2PG‐GDGT‐2 and ANME‐1b, as well as 2PG‐GDGT‐0, 2PG‐GDGT‐1, 2PG‐GDGT‐2, PG, PE, and the newly discovered HgluA‐archaeol and Bathyarchaeota. Although we cannot definitively link these lipid biomarkers with the above‐mentioned uncultured archaeal groups, these results shed light on the high diversity of IPL biomarkers of the Black Sea water column and provide additional clues on the preferential niches of the uncultured archaeal groups detected which will aid in future cultivation efforts. Finally, our results point to an important role of Bathyarchaeota and DPANN Woesearchaeota groups in the euxinic waters of the Black Sea. Future studies based on full genome metagenomic analyses are likely to unravel their metabolic and membrane lipid potential in this unique ecosystem.

## CONFLICT OF INTERESTS

The authors declare that they have no conflict of interests.
