# Supplementary material for: A combined lipidomic and 16S rRNA gene amplicon sequencing approach reveals archaeal sources of intact polar lipids in the stratified Black Sea water column
Source: Geobiology. 2018 Oct 3;17(1):91–109. doi: 10.1111/gbi.12316 (PMC6586073; doi:10.1111/gbi.12316)
Supplement: Supplementary file 1 [file GBI-17-91-s001.pdf]

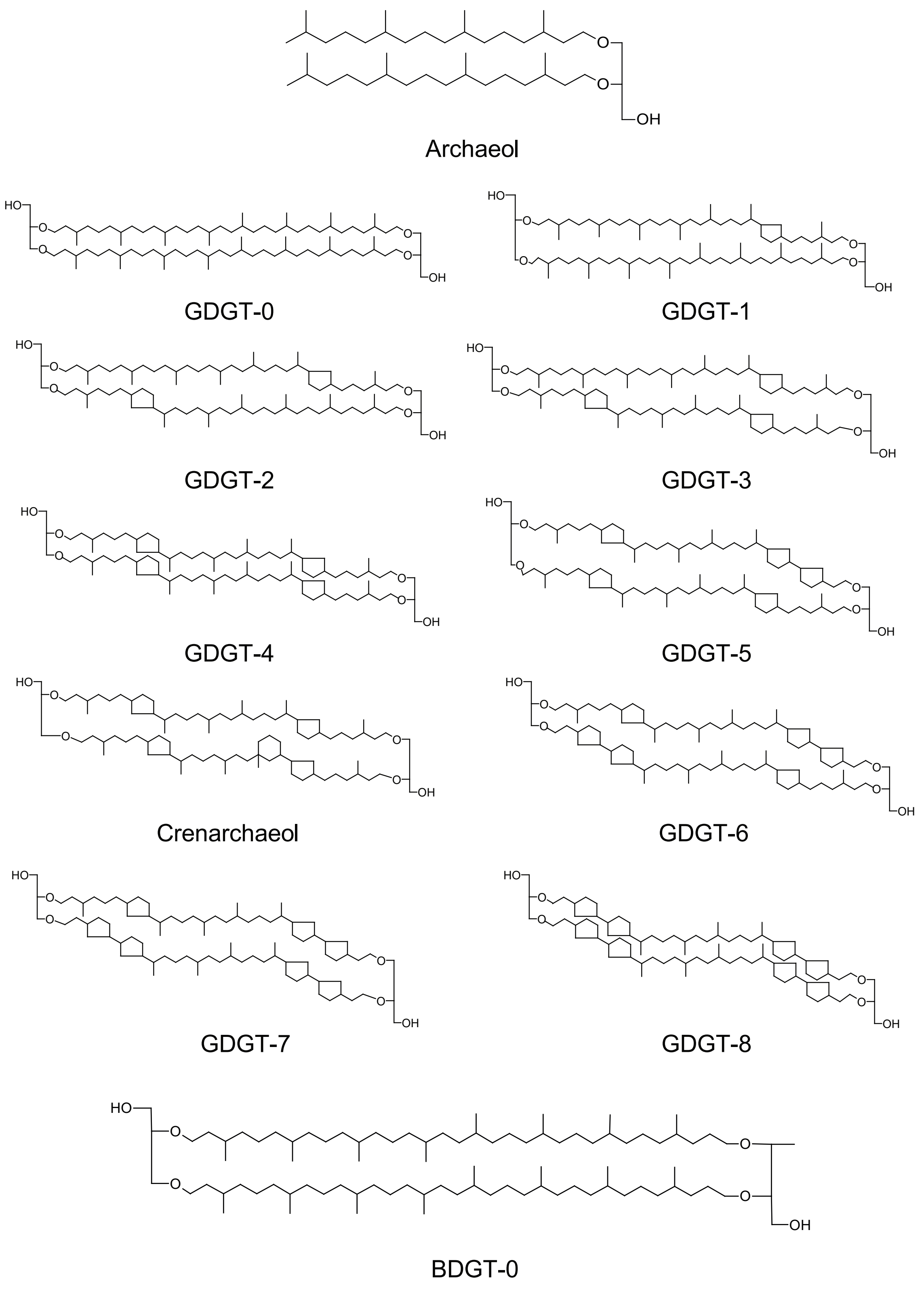

Figure S1. Structures of the archaeal membrane core lipids (CL), including those of phytanyl glycerol diether (archaeol), glycerol dibiphytanyl glycerol tetraethers (GDGTs) with 0 to 8 rings and butane-triol dibiphytanyl glycerol tetraether with 0 rings (BDGT-0).

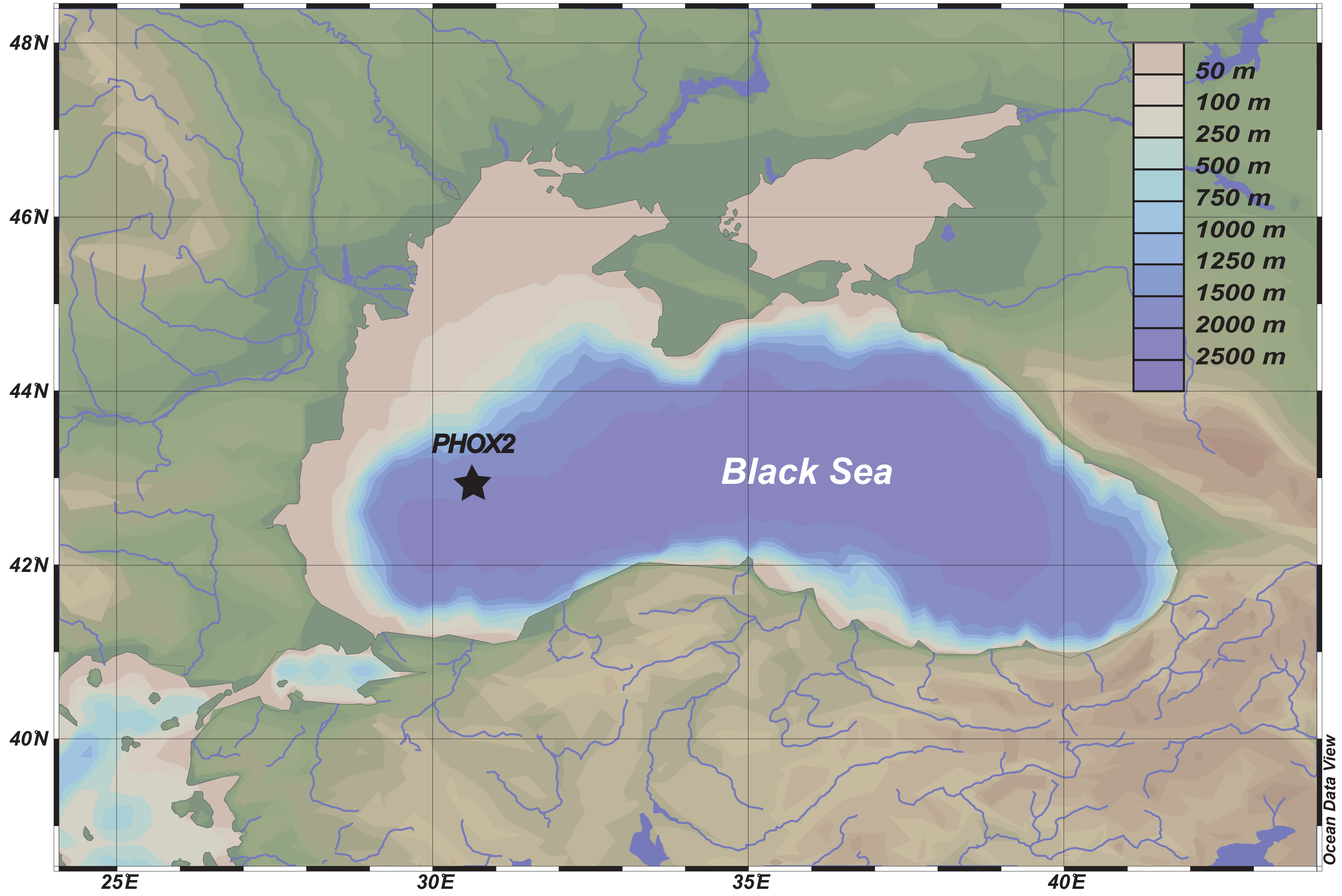

Figure S2. Map of the sampling area in the Black Sea, during the Phoxy cruise (June-July 2013). Station PHOX2, located at 42.9N and 30.7E in the western gyre, was sampled at high depth resolution for suspended particulate matter (SPM) by employing three *in situ* pump system devices.

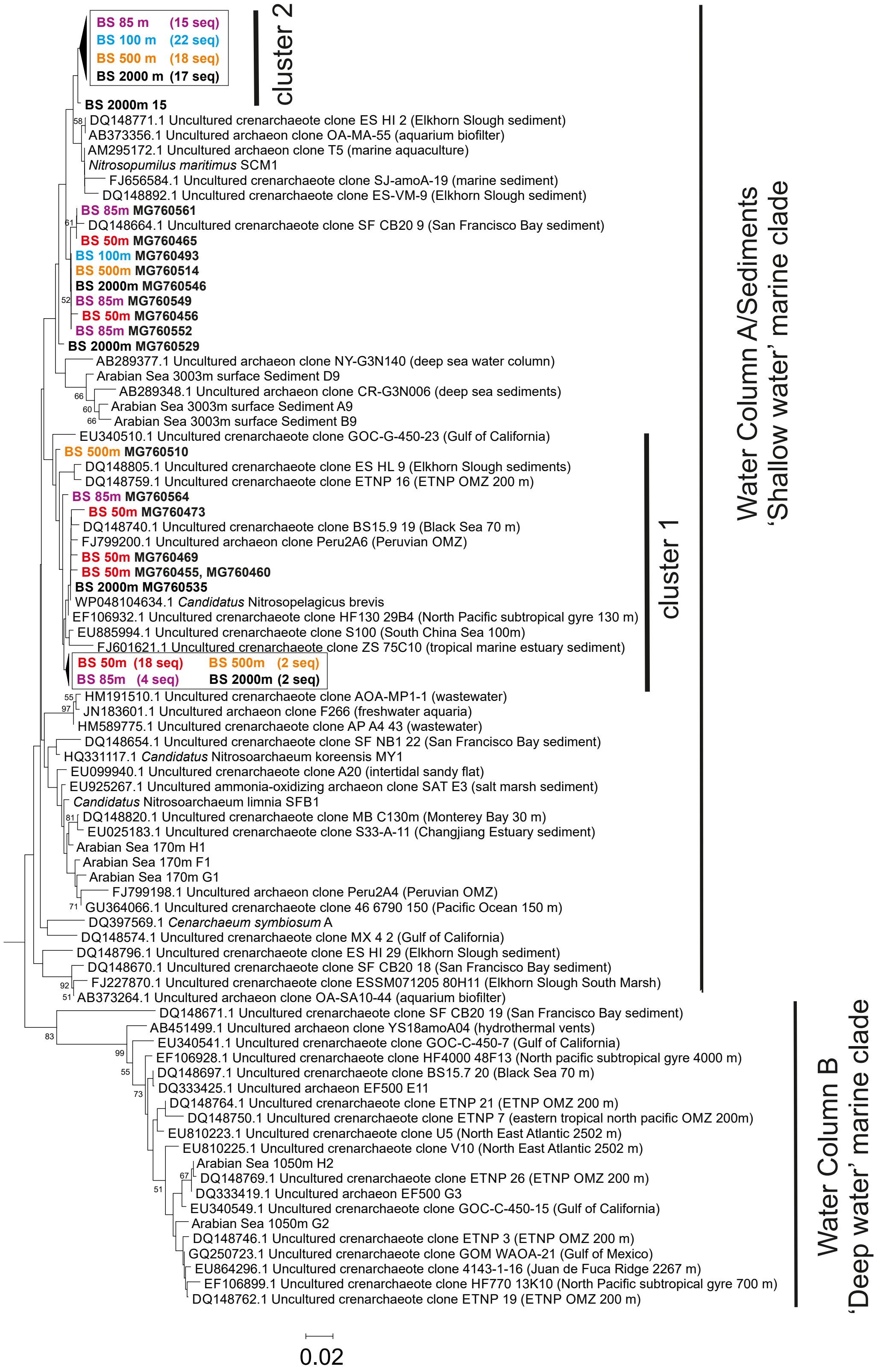

Figure S3. Neighbor-joining tree of *amoA* protein sequences recovered from the Black Sea SPM at 50 m, 85, 100, 500, and 2000 m, constructed with the Neighbor-Joining method (Saitou & Nei, 1987). Scale bar indicates 2% sequence dissimilarity. Clusters of Water column A/Sediments ('shallow water' marine clade) and B ('deep water" marine clade) of the *amoA* gene were defined by Francis *et al*. (2005). The evolutionary distances were computed using the Poisson correction method with a bootstrap test of 1,000 replicates (values higher than 50% are shown on the branches). The analysis involved 188 amino acid sequences and a total of 211 positions. The number of sequences recovered at each depth is reported. Color code: 50 m (red), 85 m (purple), 100 m (blue), 500 m (orange), 2000 m (black bold).

(a)

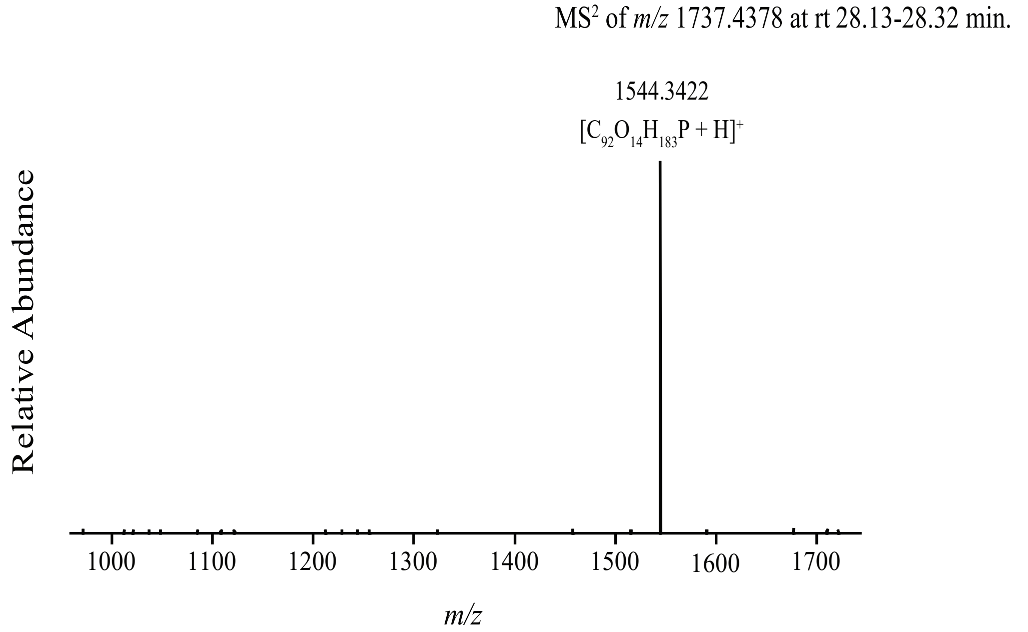

(b)

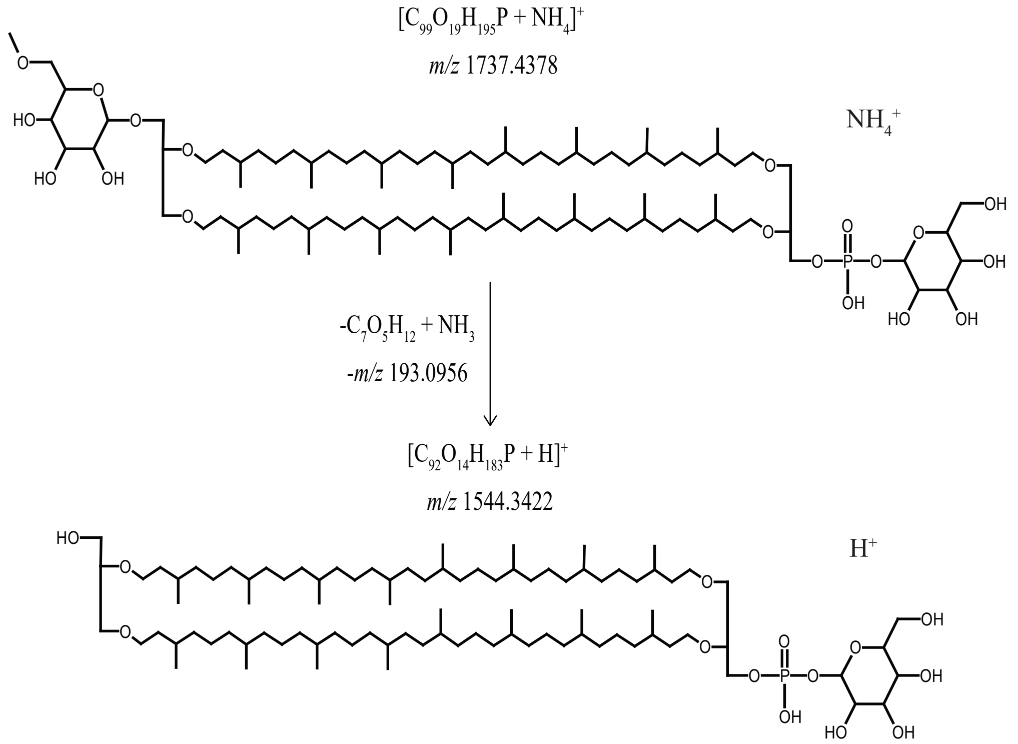

(c)

Compound	Elemental composition	Exact mass (*m/z*)[a]	Accuracy (ppm)[b]
Methoxyhexose phosphohexose-GDGT-0	$C_{99}O_{19}H_{195}P$ $[M + NH_4]^+$	1737.4368	0.6
Loss of methoxyhexose + NH_3	$C_7O_5H_{12}$	193.0950	3.0
Phosphohexose-GDGT-0	$C_{92}O_{14}H_{183}P$ $[M + H]^+$	**1544.3418**	0.2

[a] Diagnostic ions in bold indicate the major base peak observed in MS^2 spectra.

[b] The 3 ppm (parts per million) range was used as a measure of high-confidence molecular formula assignement.

Figure S4. Identification of the IPL methoxyhexose phosphohexose-GDGT-0 (i.e. MeHPH-GDGT-0) detected in the Black Sea SPM (1000 m) by LC/orbitrap-MS. (a) MS^2 fragmentation (stepped normalized collision energies 15, 22.5, 30) of *m/z* 1737.4378. (b) Proposed fragmentation pathway of the MeHPH-GDGT-0. (c) Table of the accurate masses and accuracy for MeHPH-GDGT-0 and diagnostic losses and fragments.

(a)

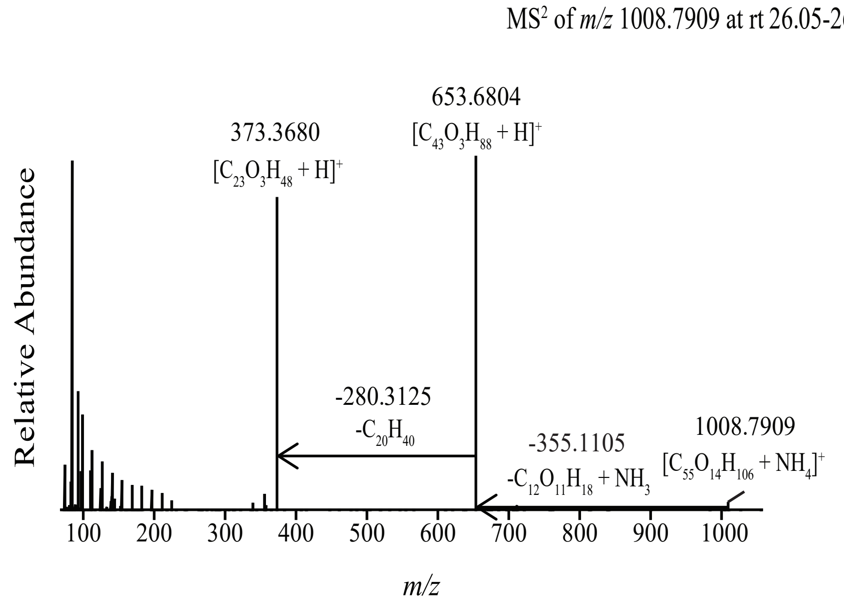

(b)

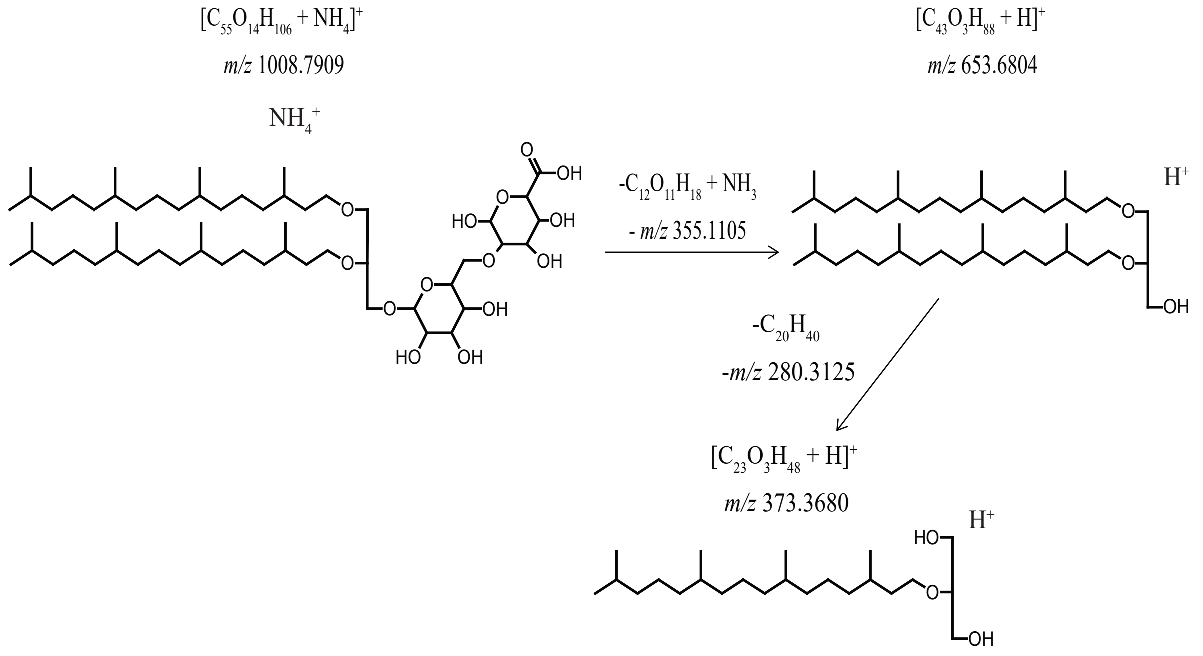

(c)

Compound	Elemental composition	Exact mass (m/z)[a]	Accuracy (ppm)[b]
HexoseGluA-archaeol	$C_{55}O_{14}H_{106}$ $[M + NH_4]^+$	1008.7921	-1.2
Loss of hexoseGluA + NH_3	$C_{12}O_{11}H_{18}$	355.1109	-1.2
Loss of phytanyl - 1 H	$C_{20}H_{40}$	280.3125	0.2
Archaeol	$C_{43}O_3H_{88}$ $[M + H]^+$	**653.6806**	-0.3
Phytanyl glycerol	$C_{23}O_3H_{48}$ $[M + H]^+$	**373.3676**	0.9

[a] Diagnostic ions in bold indicate the major base peak observed in MS^2 spectra.

[b] The 3 ppm (parts per million) range was used as a measure of high-confidence molecular formula assignement.

Figure S5. Identification of the IPL monohexose glucuronic acid-archaeol (i.e. MHgluA-archaeol) detected in the Black Sea SPM (1000 m) by LC/orbitrap-MS. (a) MS^2 fragmentation (stepped normalized collision energies 15, 22.5, 30) of *m/z* 1008.7909. (b) Proposed fragmentation pathway of the MHgluA-archaeol. (c) Table of the accurate masses and accuracy for MHgluA-archaeol and diagnostic losses and fragments.

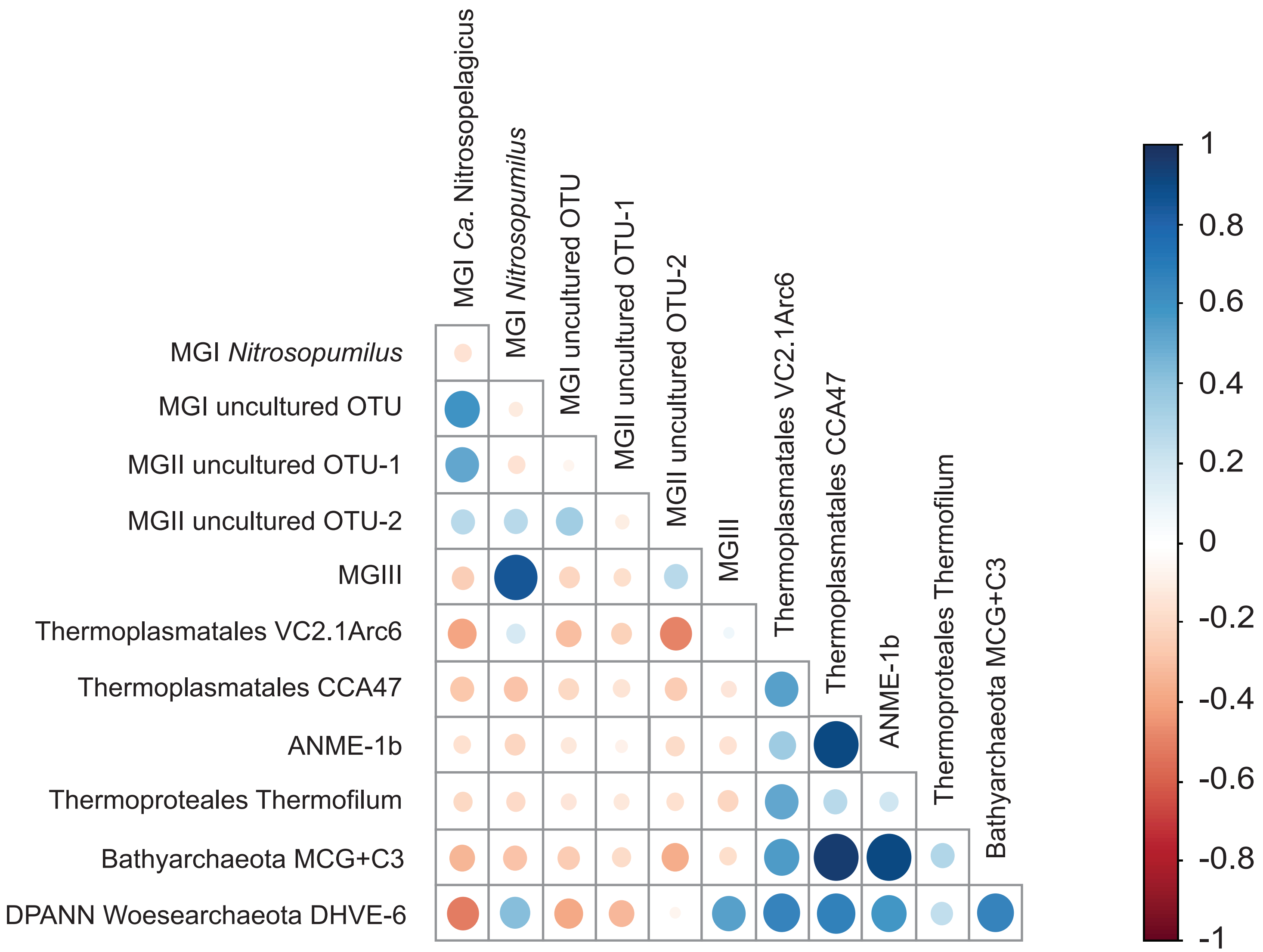

Figure S6. Dot plot of the correlation matrix obtained by applying a Pearson analysis to the total archaeal 16S rRNA gene reads (copies L^{-1}) of the archaeal groups detected across the Black Sea water column at station PHOX2. The size of the dot and the intensity of its color relate to the degree of correlation. Dark blue corresponds to r values of +1, indicating a strong positive linear correlation between archaeal groups; white corresponds to r values of 0, indicating that no correlation exists; dark red corresponds to r values of -1, indicating a strong negative linear correlation.

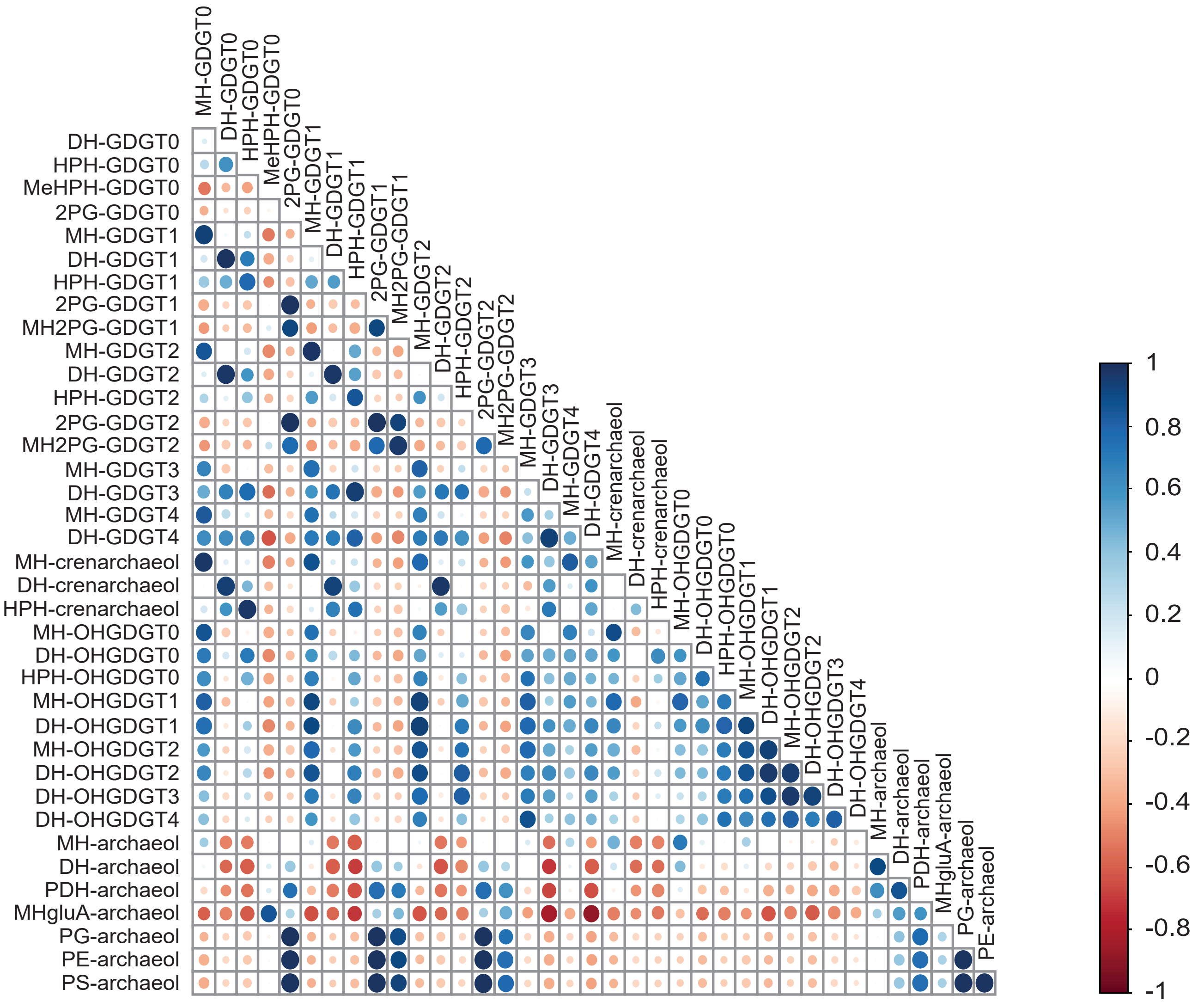

Figure S7. Dot plot of the correlation matrix obtained by applying a Pearson analysis to the absolute abundances of the archaeal IPLs (response units per Liter; r.u. L^{-1}) detected in the Black Sea water column at station PHOX2. The size of the dot and the intensity of its color relate to the degree of correlation. Dark blue corresponds to r values of +1, indicating a strong positive linear correlation between the concentrations of the archaeal IPLs; white corresponds to r values of 0, indicating that no correlation exists; dark red corresponds to r values of -1, indicating a strong negative linear correlation.
